# Osteoarthritis and fibroblast activation: an imaging study assessing feasibility and reliability of whole-body ^68^ Ga-FAPI PET-CT for structural osteoarthritis evaluation

**DOI:** 10.1007/s00256-026-05354-5

**Published:** 2026-08-31

**Authors:** Anna Katharina Roemer, Rafael Heiss, Christian Schmidkonz, Jamie E. Collins, Maria Gabriella Raimondo, Michael Beck, Ali Guermazi, Tobias Bäuerle, Georg Schett, Michael Uder, Torsten Kuwert, Andreas Ramming, Frank W. Roemer, Alina Mihaela Ramming, Armin Atzinger

**Affiliations:** 1 Department of Radiology, Universitätsklinikum Erlangen & Friedrich Alexander Universität Erlangen-Nürnberg (FAU), Erlangen, Germany; 2 Department of Nuclear Medicine, Universitätsklinikum Erlangen & Friedrich Alexander Universität Erlangen-Nürnberg (FAU), Erlangen, Germany; 3 Institute for Medical Engineering, Ostbayerische Technische Hochschule Amberg Weiden, Amberg, Germany; 4 Orthopaedic and Arthritis Center for Outcomes Research (OrACORe) and Policy and Innovation EValuation in Orthopaedic Treatments (PIVOT) Center, Department of Orthopaedic Surgery, Brigham and Women’s Hospital, Harvard Medical School, Boston, MA USA; 5 Department of Internal Medicine 3, Rheumatology & Immunology, Universitätsklinikum Erlangen & Friedrich Alexander Universität Erlangen-Nürnberg (FAU), Erlangen, Germany; 6 Quantitative Imaging Center, Department of Radiology, Chobanian & Avedisian School of Medicine, Boston University, Boston, MA USA; 7 Department of Radiology, VA Boston Healthcare System, West Roxbury, MA USA; 8 Department of Diagnostic and Interventional Radiology, Universitätsmedizin Mainz & Johannes Gutenberg-Universität Mainz, Mainz, Germany

**Keywords:** Osteoarthritis, Computed tomography, Positron emission tomography, Fibroblast, ^68^ Ga-FAPI PET-CT

## Abstract

**Objective:**

Primary aim was to evaluate whether presence of osteoarthritis (OA), as assessed by ordinal grading on whole-body computed tomography (CT), is associated with ^68^ Ga fibroblast activation protein inhibitor (FAPI) positron emission tomography (PET) tracer uptake as a measure of fibroblastic activation. Secondary aim was to evaluate whether OA disease severity is positively correlated with increased tracer uptake and to evaluate reliability.

**Material and methods:**

In a retrospective study design, patients who had undergone ^68^ Ga-FAPI PET-CT for a spectrum of clinical reasons were included. Whole-body CT was assessed for OA using the OsteoArthritis Computed Tomography‐Score in multiple joints and the spine. Maximum standard uptake value (SUV_max_) was determined correspondingly. Logistic regression and correlation analyses were used to describe associations between structural OA and ^68^ Ga-FAPI PET activity.

**Results:**

Fifty-four patients were included. Presence of OA in the acromioclavicular joints (ACJ) was associated with odds of SUV_max_ being in the highest tertile. Increased odds were seen for one location of the cervical spine (OR 5.7, 95% confidence interval [1.6, 20.4]) and both locations of the lumbar spine (OR 8.3, 95% confidence interval [1.5, 46.6]; 5.0, 95% confidence interval [1.3, 18.7]). Moderate correlations were observed for OA severity and ^68^ Ga-FAPI PET uptake for most joints and spine. Reliability for OA scoring (overall percent agreement) ranged from 40 to 100% (intra-reader) and from 66.3 to 100% (inter-reader). Mean intraclass correlation coefficients for SUV_max_ were 0.76 and 0.82, respectively.

**Conclusion:**

Presence of OA was associated with increased SUV_max_ uptake for the ACJ and the cervical and lumbar spine. In this feasibility study, CT-based OA scoring and ^68^ Ga-FAPI PET SUV_max_ determinations could be achieved with good reliability.

**Supplementary Information:**

The online version contains supplementary material available at https://doi.org/10.1007/s00256-026-05354-5.

## Introduction

Osteoarthritis (OA) is the most common type of arthritis. It can involve almost any joint but typically affects the hands, knees, hips, and feet [1]. It is characterized by pathologic changes in cartilage, bone, synovium, ligament, muscle, and periarticular fat, leading to joint dysfunction, pain, stiffness, and functional limitations [2]. As multiple metabolically active tissues are relevant for disease onset and progression of OA including the synovium and subchondral bone, there has been a new interest in the application of metabolic imaging using positron emission tomography (PET) to OA reflecting metabolic activity involved in the disease process [3]. PET offers unique abilities to provide quantitative information about molecular and metabolic activity, which are thought to be potential targets of disease-modifying OA therapies [4].

Early studies evaluating [18F] fluoro-d-glucose PET (^18^F-FDG PET) have shown marked glucose uptake in rheumatoid arthritis (RA) joints with some OA joints also exhibiting PET positivity reflecting synovitis [5]. A positive correlation of standard uptake values (SUVs) and clinical disease severity and radiographic OA severity was shown for ^18^F-FDG PET in OA [6]. In a comparative study looking at painful and non-painful OA knee joints, glucose accumulation with increased SUVs was seen particularly in the synovium of painful joints [7]. In recent years, [18F]-sodium fluoride ^18^F-fluoride (^18^F-NaF) PET has primarily been used to study OA due to its ability to assess bone remodeling. Increased subchondral bone metabolic activity has been shown in knee regions of OA subjects with osteophytes, bone marrow lesions, and also adjacent cartilage damage on magnetic resonance imaging (MRI) [8–10]. Increased subchondral bone metabolic activity was also seen in regions of normal-appearing bone and cartilage in an OA sample compared with healthy controls [9].

More recently, the relevance of fibroblastic activation has been acknowledged for several rheumatologic diseases [11]. In  RA, fibroblast-like synoviocyte cells show significant fibroblast activation protein inhibitor (FAPI) tracer uptake in a lesion-specific manner, which correlates with clinical disease activity composite scores such as the Disease Activity Score of 28 joints (DAS28) [12]. Furthermore, ^68^ Ga-FAPI PET demonstrated a greater number of joints affected and increased concentration of tracer uptake compared with fluorine ^18^F-FDG PET [13]. While OA is considered a primary non-inflammatory disorder, phases of marked synovial activation are commonly seen during the disease course, which drives clinical and structural progression [14]. In OA, pro-inflammatory factors are responsible for the progressive destruction and remodeling of the joint through the stimulation of matrix-degrading enzymes, including the matrix metalloproteinases [15, 16]. Those pro-inflammatory mediators and anabolic factors are produced locally by the cells within the affected tissues including the articular chondrocytes, synovial fibroblasts, and immune cells in the synovium [17]. The role of fibroblastic activity and quantification in an in vivo human context for disease onset and progression of OA to date is largely unclear. A recent study by Spektor and colleagues reported on patients who underwent ^68^ Ga-FAPI PET-computed tomography (CT) for oncologic reasons and assessed shoulder and hip joints for structural OA and corresponding FAPI tracer uptake [18]. Authors reported that FAPI uptake in joints is frequent and was observed in presence as well as in absence of joint degeneration. However, authors did not report on other joints commonly affected by OA, such as the thumb base, fingers, or knees, and also did not include vertebral assessment. Furthermore, reliability of the ordinal scoring and quantitative assessments was not reported in that study.

Thus, the primary aim of this feasibility study was to assess whether presence of OA, as evaluated by multi-joint, multi-anatomy ordinal grading on whole-body CT (using the CT component of ^68^ Ga-FAPI PET-CT), is associated with ^68^ Ga-FAPI PET tracer uptake (SUV_max_) as a measure of fibroblastic activity. Secondary aims were to evaluate whether OA disease severity is positively correlated with increased tracer uptake and to determine inter- and intra-reader reliability of ordinal whole-body OA assessment on CT and of quantitative SUV_max_ measurements.

## Methods

### Patient sample and inclusion

In this single-center retrospective cross-sectional study design, we included adult patients who had undergone ^68^ Ga-FAPI PET-CT for a spectrum of clinical reasons between March 2018 and June 2024. Included were all available patients who had consented to their imaging data being used for retrospective analysis. The study was approved by the Ethics Committee of the Friedrich-Alexander-Universität (FAU) Erlangen-Nürnberg (#23–359-Bp).

Main reasons for referral were (1) assessment of structural disease activity in patients with a known inflammatory rheumatologic disorder affecting synovial joints like RA, spondylarthritis (SpA), or psoriatic arthritis (PsA), or (2) patients who were imaged for work-up of a suspected inflammatory disorder, who subsequently did not fulfill diagnostic disease criteria or underwent ^68^ Ga-FAPI PET-CT for staging of pancreatic cancer. Acute or subacute musculoskeletal trauma (within the last 6 months) was an exclusion criterion. We grouped these patients into three distinct subgroups: (1) those with a diagnosed inflammatory disorder and no concomitant OA (i.e., “inflamed group”), (2) those with a diagnosed inflammatory disorder and concomitant OA (i.e., “mixed group”), and (3) those with no inflammatory disorder (i.e., “non-inflamed group”). The differentiation between groups 1 and 2 was based on the presence or absence of at least 3 joints and/or spine levels with at least early-moderate OA (i.e., at least a definite osteophyte or definite disc space narrowing) on screening the CT images by one of the last authors (A.M.R.).

### ^68^ Ga-FAPI PET-CT acquisition

From March 2018 until May 2022, ^68^ Ga-FAPI PET-CT scans were conducted with a Biograph mCT 40 PET-CT system (Siemens Healthineers, Erlangen, Germany). From June 2022 until December 2024, PET examinations were conducted on a Biograph Vision 600 (Siemens Healthineers, Erlangen, Germany) system. Patients received a median dose of 119 MBq (interquartile range (IQR) 70.2 MBq) of ^68^ Ga-FAPI-46, with a median body weight of 71 kg (IQR 17.8 kg). The median time from injection to imaging was 60 min (IQR ± 36.5 min). Most scans covered the region from the orbitae to the toes, with additional separate imaging of the hands for most patients (10 min per bed position). For some scans, particularly in group 3, the knees, hands, elbows, or ankles were not assessable as these were not or not sufficiently anatomically covered. PET data were corrected for random and scattered coincidences, decay, and attenuation. For attenuation correction, a low-dose CT over the same body area was used (100 kV, 40 ref mAs). All corrections and reconstructions were obtained using the PET-CT manufacturer’s software. All data were analyzed using Syngo.via software (Siemens Molecular Imaging, Hoffman Estates, IL, USA).

### Image assessment

The CT component of the PET-CT was evaluated using the OsteoArthritis Computed Tomography‐Score (OACT‐Score), which comprises ordinal scoring of multiple joints and the spine [19]. In addition, hands (whenever visualized and assessable) were evaluated according to Osteoarthritis Research Society International (OARSI) atlas-based grading [20]. The elbows and ankles were evaluated using a simplified grading of osteophyte presence and joint space narrowing aligned with Kellgren–Lawrence grading [21] as follows:

The acromioclavicular joint was scored from 0 to 3 according to the OACT-Score as follows: 0 = no osteophytes, no joint space narrowing (JSN); 1 = lipping and/or possible JSN; 2 = definite osteophytes and/or JSN; 3 = definite osteophytes and/or JSN and sclerosis and/or cysts and/or bony deformities. The glenohumeral joint was assessed from 0 to 3 according to the OACT-Score as follows: 0 = no osteophyte or definite JSN; 1 = osteophyte (inferior, anterior, posterior humeral, and glenoidal) measured less than 3 mm; 2 = osteophyte measured between 3 and 7 mm; 3 = osteophyte measured more than 7 mm, definite JSN and/or irregularity. Elbows were assessed from 0 to 3 for osteophyte size and JSN separately. Based on these scores, a composite score was defined as 0/1 = 0 (no OA), 2/3 = 1 (mild-moderate OA), and 4–6 = 2 (severe OA). Hand joints were scored for the carpometacarpal joint D1, the metacarpophalangeal joint D1, the distal interphalangeal joints D2–D5, the interphalangeal joint D1, and the proximal interphalangeal joints D2–D5. Osteophytes and JSN were scored from 0 to 3, respectively, according to the OARSI atlas, and scores were summed to a maximum of 6. As for the elbows, grades were defined as 0/1 = 0 (no OA), 2/3 = 1 (mild-moderate OA), and 4–6 = 2 (severe OA). The hips were scored according to the OACT-Score for JSN, 0–3; osteophyte size, 0–3; and presence/absence of subchondral cysts, for a total of 0–7. As suggested by Gielis et al. [19], the scores were converted to 0/1 = grade 0 (no OA), 2/3 = grade 1 (mild OA), 4/5 = grade 2 (mild-moderate OA), and 6/7 = grade 3 (severe OA). The knees were scored in identical fashion as the hips for the medial and lateral compartment separately, and scores were converted accordingly. The patellofemoral joint was not part of the assessment. The ankles were scored with a composite score from 0 to 3 adapted from Kellgren–Lawrence grading with 0 = no or minimal osteophytes, 1 = definite osteophyte, 2 = definite joint space narrowing, and 3 = bone-on-bone appearance. The spine was assessed according to the OACT-Score. The two most affected levels were determined for the cervical, thoracic, and lumbar spine, and three features were assessed: disc space narrowing (DSN) from 0 to 3, sclerosis and endplate irregularity (SCIR) presence or absence, and anterior or posterior osteophytes from 0 to 3. A composite score was created according to Gielis et al. [19] as DSN score × 2 (0–6), osteophyte score 0–3, and SCIR 0–1 for a total of 0–10. Grades 0–2 were defined as grade 0, grades 3–5 as grade 1, grades 6–8 as grade 2, and grades 9 and 10 as grade 3. The facet joints were not part of the assessment for reasons of feasibility.

**Fig. 1:**
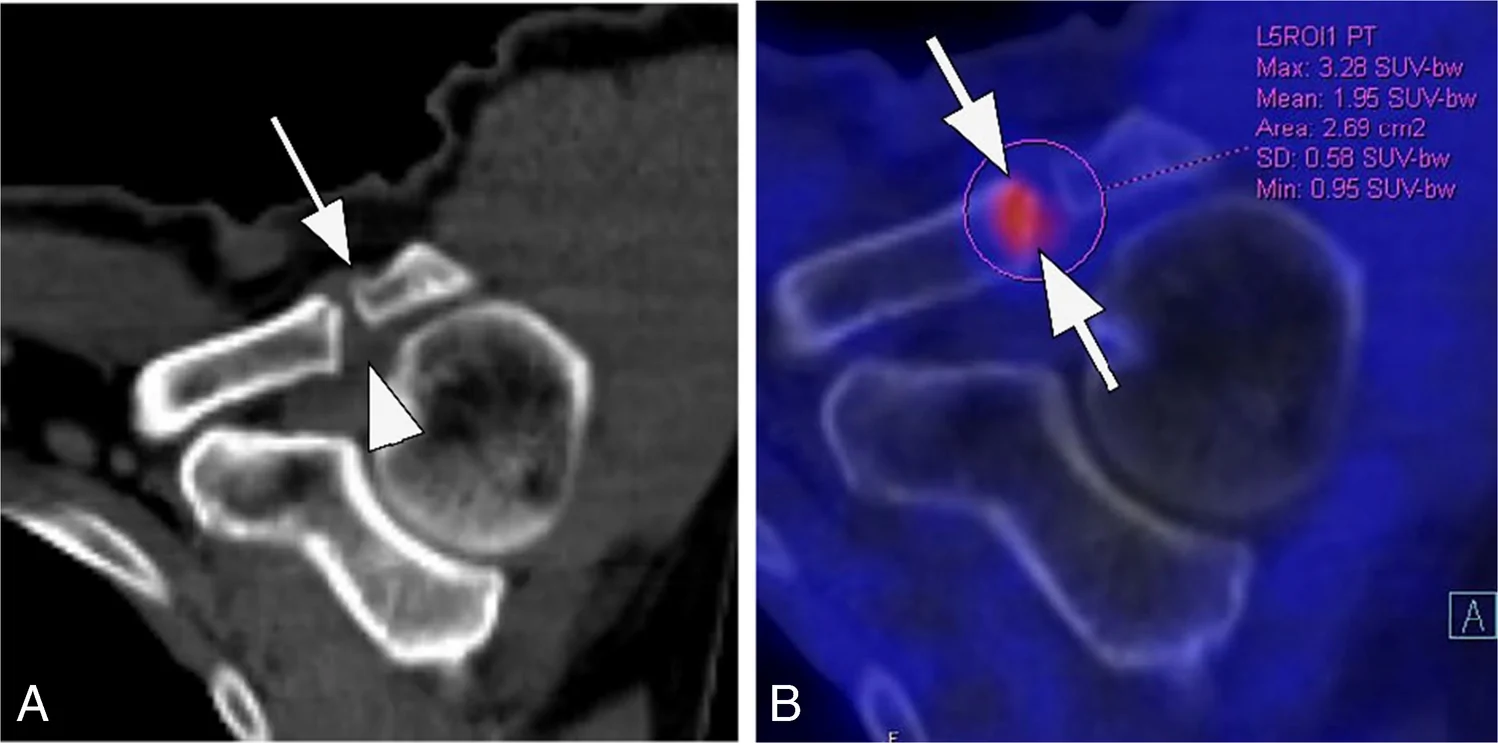
Acromioclavicular joint. Sixty-four-year-old male patient with no history of inflammatory disorder. FAPI tracer uptake was commonly observed in all disease categories. **a** CT image shows minor signs of osteoarthritis at the left acromioclavicular joint with an osteophyte (arrows) and capsular thickening (arrowhead). **b** Corresponding fusion image shows moderate FAPI tracer uptake (SUV_max_ 3.28) at the lateral clavicle without corresponding structural pathology on CT (arrows), a finding of unknown clinical or structural relevance reflecting fibroblastic activity

**Fig. 2:**
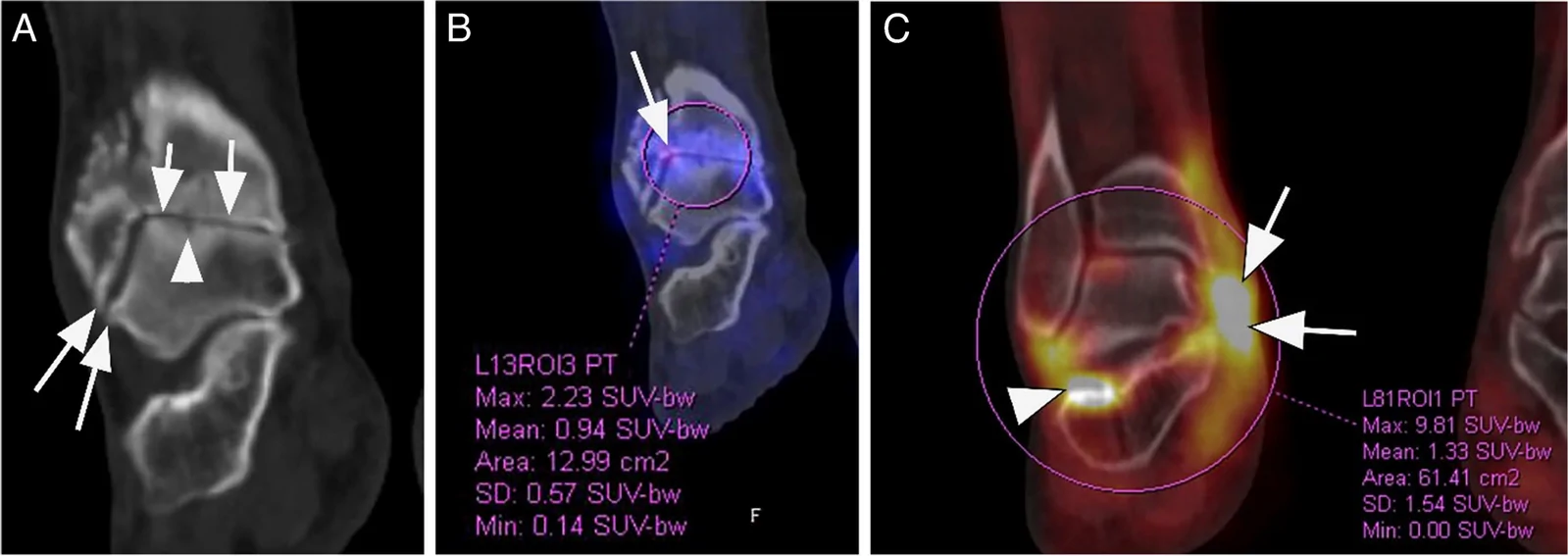
Ankle. Fifty-seven-year-old male patient with absence of known inflammatory arthritis. **a** Coronal reformation of CT shows marked joint space narrowing of the central tibiotalar joint (short arrows) and a small subchondral cyst (arrowhead) characteristic of advanced OA. In addition, there are osteophytes at the medial tibiotalar margin (large arrows). **b** Corresponding coronal fusion image with FAPI PET shows mild tracer uptake (SUV of 2.23) at the medial talar shoulder and corresponding tibial joint in a subchondral location (arrow). **c** Forty-eight-year-old female patient with known spondylarthritis. While there are no obvious signs of OA, there is marked tracer uptake around the posterior tibialis tendon (arrows) and also in the inferior aspects of the sinus tarsi (arrowhead), most likely due to tenosynovitis and entheseal inflammation

**Fig. 3:**
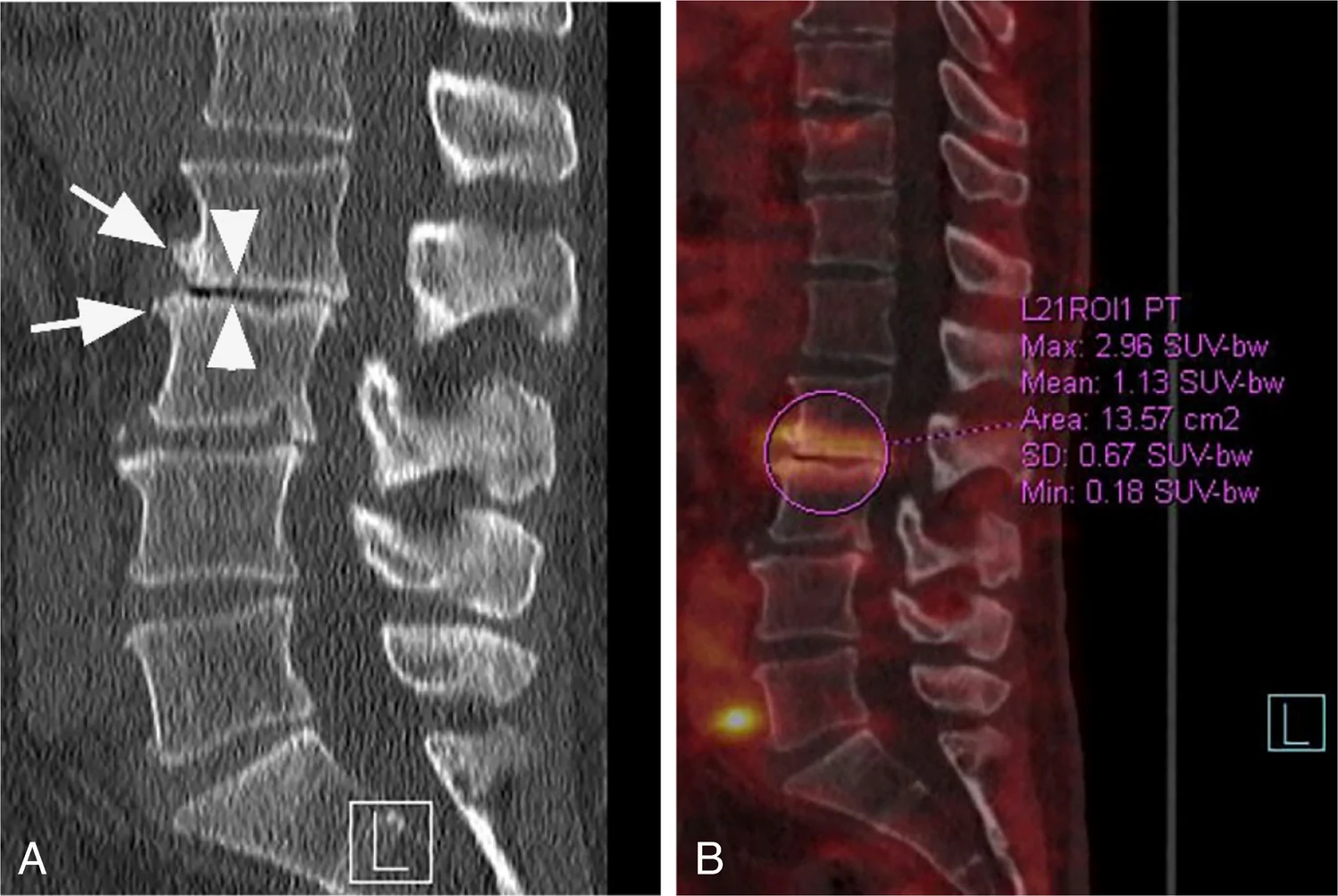
Spine. Sixty-year-old female patient without inflammatory disorder. **a** Sagittal reformat of the lumbar spine shows marked degenerative disc disease at the L2/L3 level. There is marked intervertebral space narrowing with endplate irregularities, subchondral sclerosis (arrowheads), and anterior osteophytes (arrows). **b** Corresponding fusion image shows increased tracer uptake in the peridiscal location at the L2 and L3 endplates. In addition, there is anterior uptake at the location of the osteophytes. Tracer uptake was moderate with an SUV_max_ of 2.96

The PET component was assessed by region of interest (ROI) analysis on a single reformatted slice in the center of a joint or the spine, i.e., coronal for the acromioclavicular, glenohumeral, elbow, hands (11 joints for each hand), hip, knee, and ankle joints. Sagittal ROIs were reformatted for the spine. ROIs were placed in the midline at two levels of the cervical, thoracic, and lumbar spine each, where visually structural OA (i.e., anterior and posterior osteophytes, endplate irregularities, and disc space narrowing) was most severe. For the SUV_max_ measurements, circular ROIs were placed and adapted in size to the anatomy. The entire joint was covered including the subchondral bone and the periarticular soft tissues. An example of ROI placement of the acromioclavicular joint is shown in Fig. 1 and that of the ankle in Fig. 2. Lumbar spine assessment and ROI placement are depicted in Fig. 3.

Altogether, 22 hand joints, 10 other joints, and 6 levels of the spine were included per patient. In cases when the anatomy was not covered by the PET-CT, “not applicable” was recorded. Screenshots were obtained for each SUV measurement for purposes of documentation. The primary assessment of all image data was performed by the first author (A.K.R.), a PhD student, following extensive training in ordinal OA grading and SUV evaluation The primary reader was trained by 2 experienced musculoskeletal (MSK) radiologists with 23 years and 7 years of experience in standardized OA assessment (F.W.R. and R.H.) and a nuclear medicine expert with 6 years of experience of standardized image assessment (A.A.). The training comprised 20 h of supervised training using 10 patients from the current sample and another randomly chosen 15 patients who had undergone ^18^F-FDG PET-CT not part of the dataset used for this analysis. All data were analyzed using Syngo.via software (Siemens Molecular Imaging, Hoffman Estates, IL, USA).

Intra- and inter-reader reliability assessments were performed for 15 patients, with 5 patients randomly selected from each of the 3 subgroups. Inter-reader reliability for CT-based ordinal scoring using the OACT-Score was performed by a second reader (A.G.) with 25 years of experience in standardized semiquantitative ordinal scoring of structural OA. Inter-reader reliability for the SUV evaluation was performed by the same MSK radiologist (R.H.) who had also been part of the training of the primary reader.

### Analytic approach

Descriptive statistics were used to plot OA disease severity based on CT assessment for each joint and spine location against the corresponding SUV_max_ measurements for the three subgroups and the entire sample. Logistic regression analyses were performed for presence of structural OA (using absence of OA as the referent) and the cross-sectional odds to having high SUV_max_ uptake. High SUV_max_ uptake was defined as the highest tertile of that joint/location’s SUV_max_ distribution across the full sample.

Spearman correlations were applied to evaluate associations between CT-based OA disease severity (ordinal) and concomitant tracer uptake. Concerning interpretation of the correlation coefficients, 0–0.3 were considered as small, 0.31–0.5 as medium, and ≥ 0.5 as large correlations [22]. Analyses were performed at the joint/location level, with a given statistical model including only one observation per participant.

Reliability was determined using weighted (w) kappa statistics. Cicchetti–Allison weights were used to compute the weighted kappa coefficients. Point estimates of w-kappa values were interpreted according to the suggestions of Landis and Koch as < 0 = no agreement, 0.01–0.20 = slight, 0.21–0.40 = fair, 0.41–0.60 = moderate, 0.61–0.80 = substantial, and 0.81–1.00 almost perfect agreement [23]. As the low prevalence of certain features may adversely affect the kappa results, the overall percent agreement was calculated in addition. For the quantitative SUV_max_ measurements, intraclass correlation coefficients (ICC) and Pearson correlation coefficients were determined. For ICC interpretation, we used the guidelines by Koo and Li categorizing them as poor (< 0.50), moderate (0.50–0.75), good (0.75–0.90), and excellent (> 0.90) [24]. Reliability analysis for some joint sites included > 1 joint per participant. For these joints, we used cluster bootstrapping to generate 95% confidence intervals to account for within-participant correlation.

All statistical analyses were performed by a senior biostatistician with 12 years of experience in data analysis of structural OA (J.E.C.) using SAS v9.4 (SAS Institute, Cary, NC, USA). No adjustment for multiple testing was used, given the exploratory nature of this study [25].

## Results

Altogether, 54 patients were included: 25 in the inflamed group, 11 in the mixed group, and 18 in the non-inflamed group. Mean age for the entire sample was 47.7 years (standard deviation (SD) ± 15.9, range 21–86 years). Mean age in the inflamed group was 38.1 years (SD ± 10.7, range 21–62 years); in the mixed group, it was 48.7 years (SD ± 12.7, range 29–63 years); and for the non-inflamed group, it was 63.0 years (SD ± 11.8, range 32–86 years). In the inflamed and mixed groups, 6 had a diagnosis of RA, 10 had PsA, and 20 had seronegative SpA. The majority of patients (98%) with an established diagnosis of an inflammatory disorder were treated with conventional synthetic disease-modifying antirheumatic drugs or other biologics.

**Fig. 4:**
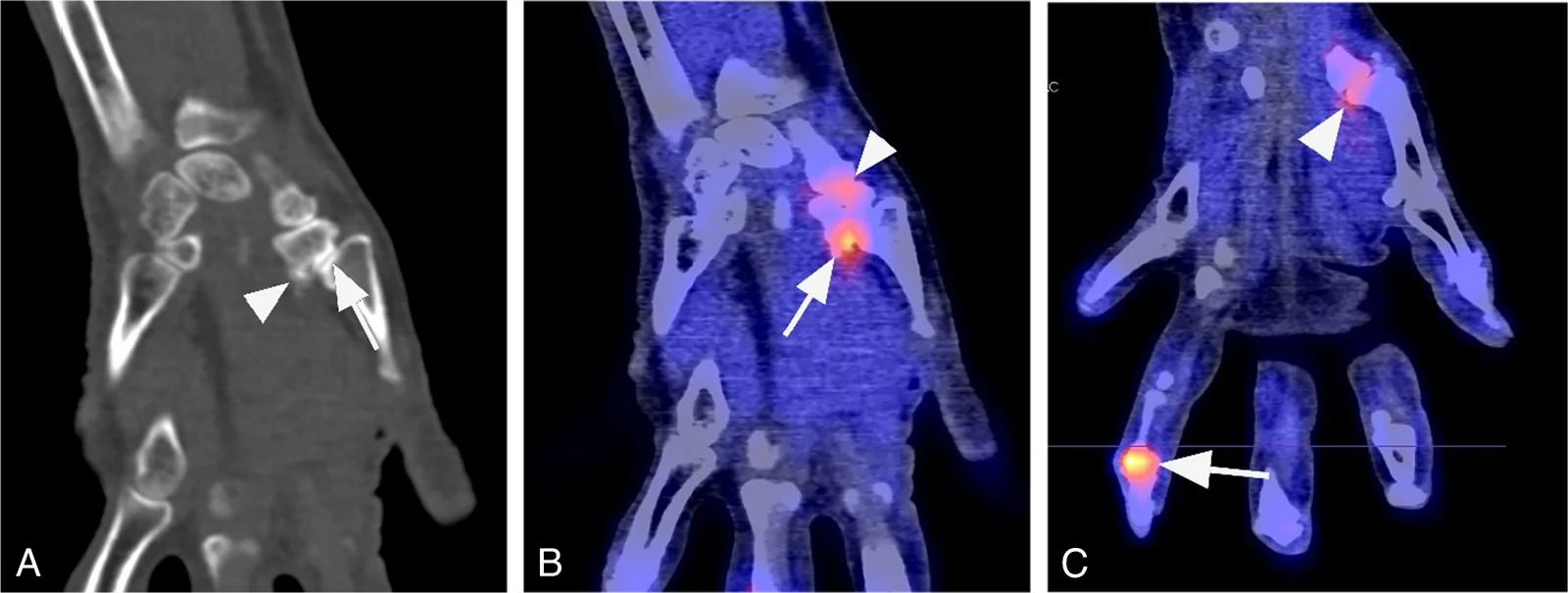
Hand. Presence of OA and possible concomitant inflammatory activity. Sixty-four-year-old female with “mixed” status (known psoriatic arthritis for 10 years and presence of structural OA). **a** Coronal CT reformation shows advanced thumb base OA with marked joint space narrowing and subchondral sclerosis (arrow). In addition, there is a definite osteophyte at the os trapezium (arrowhead). **b** Corresponding FAPI-PET fusion images show increased tracer uptake at the first CMC joint likely reflecting synovial activity (arrow). In addition, there is increased uptake also at the scaphoid-trapezial joint (arrowhead). **c** In addition to the CMC activity (arrowhead), there is also marked uptake at the distal interphalangeal joint of the second finger (arrow). As no obvious structural OA was seen in this joint, this is likely due to concomitant psoriatic arthritis disease activity

Supplementary Tables 1, 2, and 3 present the descriptive data for OA presence and severity for the entire sample and the three subgroups for the different joints and spine and corresponding SUV_max_ values. The majority of large joints in the overall sample did not exhibit structural OA (e.g., 94.9% for the right glenohumeral joint and 97.4% for the left). Most structural OA was seen in the acromioclavicular joint (47.2% for the right and 59.6% for the left). The majority of elbows and a high proportion of knees and ankles were not assessable due to missing anatomic coverage. Mean SUV_max_ values for large joints ranged from 0.37 (grade 0 left ankle non-inflammatory group) to 15.85 (grade 1 left knee medial inflammatory group). Supplementary Tables 1a and b show the descriptive data for the large joints. In only a minority of the patients, the hands were fully assessable. OA presence for the different hand joints ranged from 9.5% (interphalangeal (IP) joint D1, left hand) to 61.5% (distal interphalangeal (DIP) joints, right hand). Mean SUV_max_ for the hands ranged from 0.73 (grade 1 carpometacarpal (CMC) D1, right hand) to 5.63 (grade 3 + proximal interphalangeal (PIP) joints, left hand). Supplementary Tables 2a and b present the OA grades and SUV_max_ values for the hands. Figure 4 shows an example of increased metabolic activity with and without concomitant OA in different joints of the same hand.

The large majority of spine locations were assessable. For the overall sample, OA presence defined as grade 1 or more was observed in 31.4% (cervical 1), 52.9% (cervical 2), 61.5% (thoracic 1), 56.9% (thoracic 2), 15.1% (lumbar 1), and 24.1% (lumbar 2). Mean SUV_max_ for the overall sample ranged from 1.26 (grade 0 lumbar 2) to 3.56 (grade 2 lumbar 1). The descriptive overview of the spine results is given in Supplementary Table 3.

Table 1 presents the associations between structural OA presence (using absence of OA as the referent) and the odds of increased FAPI uptake defined as the highest tertile of mean SUV_max_ values for the entire sample. Increased odds of increased tracer uptake were observed for both acromioclavicular joints (left: odds ratio (OR) 8.9, 95% confidence interval (CI) [1.8, 44.9]; right: OR 12.5, 95% CI [3.0, 52.8]), for the spine cervical 1 location (OR 5.7, 95% CI [1.6, 20.4]), and for both lumbar spine locations (spine lumbar 1: OR 8.3, 95% CI [1.5, 46.6]; spine lumbar 2: OR 5.0, 95% CI [1.3, 18.7]).

**Table 1:** Association between presence of structural osteoarthritis and odds of high SUV_max_ (tertile 3)

Joint or anatomy	OA status^a^	FAPI PET (mean SUV_max_), *n* (%)		OR (95% CI)
		Tertiles 1 and 2	Tertile 3	
Ankle, left^b^	Absence (*n* = 27)	18 (66.7)	9 (33.3)	Reference
	Presence (*n* = 2)	1 (50)	1 (50)	2.0 (0.1, 35.8)
Ankle, right	Absence (*n* = 28)	19 (67.9)	9 (32.1)	Reference
	Presence (*n* = 1)	0 (0)	1 (100)	n/a^b^
Elbows, left	Absence (*n* = 19)	13 (68.4)	6 (31.6)	Reference
	Presence (*n* = 0)	0 (0)	0 (0)	n/a^b^
Elbows, right	Absence (*n* = 18)	12 (66.7)	6 (33.3)	Reference
	Presence (*n* = 0)	0 (0)	0 (0)	n/a^b^
Hands, DIP left	Absence (*n* = 7)	7 (100)	0 (0)	Reference
	Presence (*n* = 14)	7 (50)	7 (50)	n/a^b^
Hands, DIP right	Absence (*n* = 5)	5 (100)	0 (0)	Reference
	Presence (*n* = 16)	10 (62.5)	6 (37.5)	n/a^b^
Hands, PIP left	Absence (*n* = 9)	6 (66.7)	3 (33.3)	Reference
	Presence (*n* = 12)	8 (66.7)	4 (33.3)	1.0 (0.2, 6.3)
Hands, PIP right	Absence (*n* = 11)	9 (81.8)	2 (18.2)	Reference
	Presence (*n* = 10)	5 (50)	5 (50)	4.5 (0.6, 32.3)
Hands, CMC left	Absence (*n* = 18)	13 (72.2)	5 (27.8)	Reference
	Presence (*n* = 4)	2 (50)	2 (50)	2.6 (0.3, 23.8)
Hands, CMC right	Absence (*n* = 19)	13 (68.4)	6 (31.6)	Reference
	Presence (*n* = 3)	2 (66.7)	1 (33.3)	1.1 (0.1, 14.4)
Hands, IP left	Absence (*n* = 18)	12 (66.7)	6 (33.3)	Reference
	Presence (*n* = 2)	1 (50)	1 (50)	2.0 (0.1, 37.8)
Hands, IP right	Absence (*n* = 18)	12 (66.7)	6 (33.3)	
	Presence (*n* = 3)	2 (66.7)	1 (33.3)	1.0 (0.1, 13.4)
Hips, left	Absence (*n* = 50)	34 (68)	16 (32)	Reference
	Presence (*n* = 2)	0 (0)	2 (100)	n/a^b^
Hips, right	Absence (*n* = 48)	34 (70.8)	14 (29.2)	Reference
	Presence (*n* = 4)	1 (25)	3 (75)	7.3 (0.7, 76.2)
Knees, lateral left	Absence (*n* = 31)	21 (67.7)	10 (32.3)	Reference
	Presence (*n* = 0)	0 (0)	0 (0)	n/a^b^
Knees, lateral right	Absence (*n* = 29)	21 (72.4)	8 (27.6)	Reference
	Presence (*n* = 2)	0 (0)	2 (100)	n/a^b^
Knees, medial left	Absence (*n* = 30)	21 (70)	9 (30)	Reference
	Presence (*n* = 1)	0 (0)	1 (100)	n/a^b^
Knees, medial right	Absence (*n* = 28)	21 (75)	7 (25)	Reference
	Presence (*n* = 3)	0 (0)	3 (100)	n/a^b^
Shoulder, ACJ left	Absence (*n* = 21)	19 (90.5)	2 (9.5)	Reference
	Presence (*n* = 31)	16 (51.6)	15 (48.4)	8.9 (1.8, 44.9)
Shoulder, ACJ right	Absence (*n* = 28)	25 (89.3)	3 (10.7)	Reference
	Presence (*n* = 25)	10 (40)	15 (60)	12.5 (3.0, 52.8)
Shoulder, GHJ left	Absence (*n* = 38)	26 (68.4)	12 (31.6)	Reference
	Presence (*n* = 1)	0 (0)	1 (100)	n/a^b^
Shoulder, GHJ right	Absence (*n* = 37)	26 (70.3)	11 (29.7)	Reference
	Presence (*n* = 2)	0 (0)	2 (100)	n/a^b^
Spine, cervical 1	Absence (*n* = 35)	28 (80)	7 (20)	Reference
	Presence (*n* = 17)	7 (41.2)	10 (58.8)	5.7 (1.6, 20.4)
Spine, cervical 2	Absence (*n* = 24)	19 (79.2)	5 (20.8)	Reference
	Presence (*n* = 28)	16 (57.1)	12 (42.9)	2.9 (0.8, 9.8)
Spine, thoracic 1	Absence (*n* = 20)	15 (75)	5 (25)	Reference
	Presence (*n* = 32)	20 (62.5)	12 (37.5)	1.8 (0.5, 6.2)
Spine, thoracic 2	Absence (*n* = 22)	17 (77.3)	5 (22.7)	Reference
	Presence (*n* = 29)	17 (58.6)	12 (41.4)	2.4 (0.7, 8.3)
Spine, lumbar 1	Absence (*n* = 45)	33 (73.3)	12 (26.7)	Reference
	Presence (*n* = 8)	2 (25)	6 (75)	8.3 (1.5, 46.6)
Spine, lumbar 2	Absence (*n* = 41)	31 (75.6)	10 (24.4)	Reference
	Presence (*n* = 13)	5 (38.5)	8 (61.5)	5.0 (1.3, 18.7)

*n/a* not applicable, *ACJ* acromioclavicular joint, *GHJ* glenohumeral joint

^a^Presence of osteoarthritis defined as any non-zero score for ankle, and shoulder ACJ and GHJ; elbow, hands, hips, and knees (summed score of osteophyte size and JSN > 1); and spine (summed score of disc space narrowing sclerosis and endplate irregularity and osteophytes presence > 2)

^b^No statistical analyses possible as either referent or both groups (tertiles 1 and 2 and tertile 3) did not have ajoint or feature with osteoarthritis presence

**Fig. 5:**
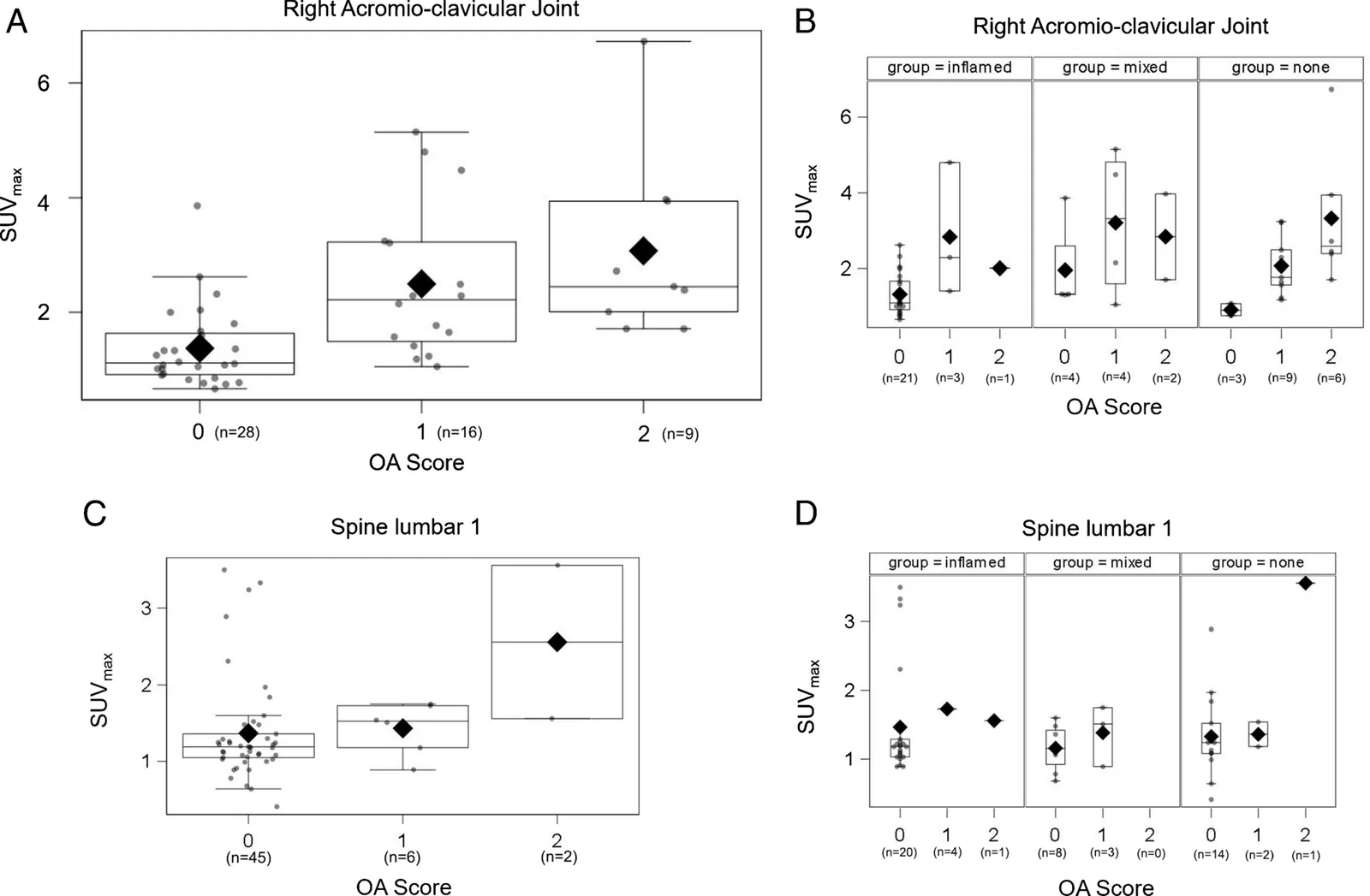
Exemplary correlation analyses of structural disease severity and tracer uptake (SUV_max_) for the right acromioclavicular joint and the lumbar 1, location of the lumbar spine. For reasons of low frequencies in the grade 3 category for both joints/locations, grades 2 and 3 were combined. **a** Right acromioclavicular joint entire sample (correlation estimate 0.61, 95% confidence interval [0.41, 0.76]). **b** Right acromioclavicular joint stratified by subgroups for inflammatory (correlation estimate 0.44, 95% CI [0.06, 0.71]; mixed: correlation estimate 0.37, 95% CI [− 0.34, 0.81]) and non-inflammatory (correlation estimate 0.68, 95% CI [0.31, 0.87]) groups. **c** Lumbar 1, location of the lumbar spine (correlation estimate 0.27, 95% confidence interval [0.004, 0.50]). **d** Lumbar 1, location of the lumbar spine for inflammatory (correlation estimate 0.30, 95% CI [− 0.11, 0.62]; mixed: correlation estimate 0.31, 95% CI [− 0.36, 0.77]) and non-inflammatory (correlation estimate 0.33, 95% CI [− 0.17, 0.70]) groups

**Fig. 6:**
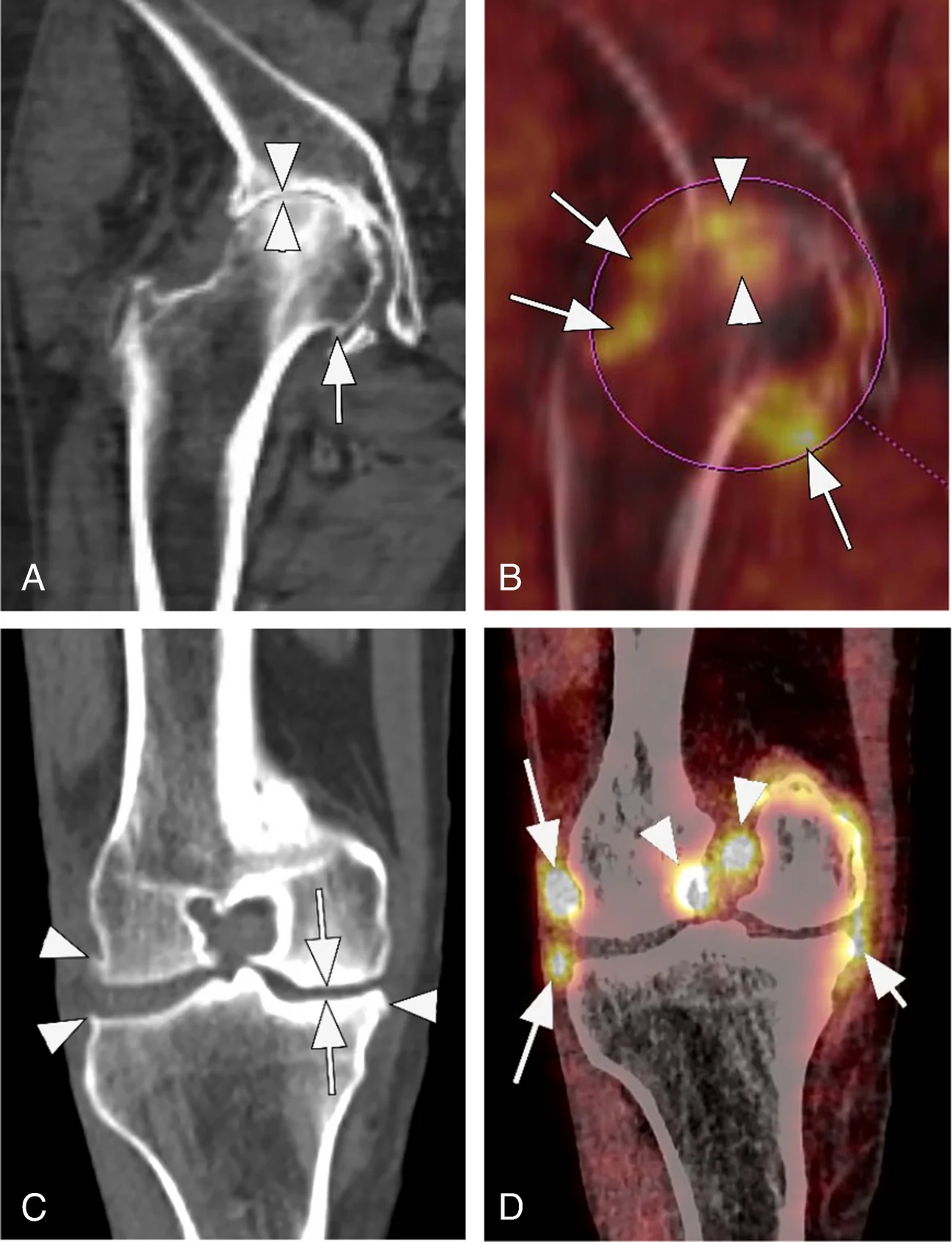
Structural OA and corresponding increased FAPI-PET tracer uptake in a patient without and with inflammatory disorder. **a** Eighty-one-year-old female without inflammatory disorder (examination was performed for evaluation of pancreatic cancer). Coronal reformation of CT shows marked joint space narrowing (arrowheads) and a small osteophyte at the inferior medial femoral head–neck junction (arrow). **b** Corresponding fusion image shows synovial tracer uptake laterally and also in an inferior-medial location (arrows). In addition, there is increased FAPI tracer uptake in a subchondral location at the acetabulum and superior femoral head (arrowheads). **c** Fifty-four-year-old male patient with known seronegative spondylarthritis. Coronal reformation of CT shows definite OA Kellgren–Lawrence grade 3 with medial joint space narrowing and sclerosis (arrows) and presence of definite osteophytes medially and laterally (arrowheads). **d** Corresponding fusion image shows marked FAPI tracer uptake lateral at the insertion of the popliteal tendon and in a perimeniscal location (long arrows). In addition, there is tracer uptake in the femoral notch around the anterior cruciate ligament and the lateral femoral condyle (arrowheads). At the medial aspect of the joint, there is tracer uptake in the perimeniscal region (short arrow). Whether these increased FAPI activity is due to OA or the inflammatory disorder is unclear

Table 2 shows the correlation analyses between structural OA disease severity and SUV_max_ tracer uptake. Most correlations were small or medium with high correlations > 0.5 seen for both acromioclavicular joints (left: OR 0.56, 95% CI [0.34, 0.73]; right: OR 0.61, 95% CI [0.41, 0.76]), left (OR 0.77, 95% CI [0.44, 0.92]) and right (OR 0.59, 95% CI [0.06, 0.87]) DIP, and right PIP (OR 0.52, 95% CI [0.06, 0.80]) joints for hands. Figure 5 shows a more detailed graphic visualization in exemplary fashion for the right acromioclavicular joint and the spine lumbar 1 location for the entire sample and the different subgroups. On an individual basis and in exemplary cases, definite or severe OA and increased tracer uptake were seen for patients with and without underlying inflammatory disease. An example of structural hip OA and marked concomitant FAPI activity in a patient without inflammatory disorder and in a different patient with knee OA and an established diagnosis of peripheral spondylarthritis and corresponding FAPI activity is shown in Fig. 6.

**Table 2:** Spearman correlations between osteoarthritis severity and tracer uptake (SUV_max_)

Joint or anatomy	Group	Number of patients	Correlation estimate	Lower 95% confidence limit	Upper 95% confidence limit
Ankle, left	All	29	0.23	− 0.15	0.55
	Inflamed	16	0.35	− 0.17	0.73
	Mixed	10	n/a^a^	n/a^a^	n/a^a^
	None	3	n/a^a^	n/a^a^	n/a^a^
Ankle, right	Overall	29	0.27	− 0.11	0.58
	Inflamed	16	n/a^a^	n/a^a^	n/a^a^
	Mixed	10	0.39	− 0.32	0.82
	None	3	n/a^a^	n/a^a^	n/a^a^
Elbows, left	Overall	19	n/a^a^	n/a^a^	n/a^a^
Elbows, right	Overall	18	n/a^a^	n/a^a^	n/a^a^
Hands (sum of 3 features), DIP left	Overall	16	0.77	0.44	0.92
	Inflamed	7	0.83	0.20	0.97
	Mixed	8	0.76	0.11	0.95
	None	1	n/a^a^	n/a^a^	n/a^a^
Hands (sum of 3 features), DIP right	Overall	13	0.59	0.06	0.87
	Inflamed	5	0.68	− 0.50	0.98
	Mixed	7	0.58	− 0.30	0.93
	None	1	n/a^a^	n/a^a^	n/a^a^
Hands (sum of 3 features), PIP left	Overall	17	0.32	− 0.19	0.70
	Inflamed	8	0.15	− 0.62	0.77
	Mixed	8	0.61	− 0.16	0.92
	None	1	n/a^a^	n/a^a^	n/a^a^
Hands (sum of 3 features), PIP right	Overall	17	0.52	0.06	0.80
	Inflamed	8	0.41	− 0.41	0.87
	Mixed	8	0.73	0.06	0.95
	None	1	n/a^a^	n/a^a^	n/a^a^
Hands, CMC left	Overall	22	0.25	− 0.19	0.61
	Inflamed	12	0.25	− 0.38	0.72
	Mixed	9	0.24	− 0.51	0.78
	None	1	n/a^a^	n/a^a^	n/a^a^
Hands, CMC right	Overall	22	− 0.15	− 0.54	0.29
	Inflamed	12	− 0.46	− 0.82	0.15
	Mixed	9	− 0.26	− 0.79	0.49
	None	1	n/a^a^	n/a^a^	n/a^a^
Hands IP D1 Left	Overall	20	0.19	− 0.28	0.58
	Inflamed	11	0.48	− 0.17	0.84
	Mixed	8	− 0.23	− 0.80	0.57
	None	1	n/a^a^	n/a^a^	n/a^a^
Hands, IP D1 right	Overall	21	0.22	− 0.23	0.59
	Inflamed	12	n/a^a^	n/a^a^	n/a^a^
	Mixed	8	− 0.05	− 0.73	0.68
	None	1	n/a^a^	n/a^a^	n/a^a^
Hips (3 features), left	Overall	52	0.33	0.06	0.55
	Inflamed	24	0.34	− 0.07	0.65
	Mixed	11	n/a^a^	n/a^a^	n/a^a^
	None	17	0.40	− 0.10	0.74
Hips (3 features), right	Overall	52	0.31	0.04	0.53
	Inflamed	24	0.27	− 0.15	0.60
	Mixed	11	0.29	− 0.38	0.76
	None	17	0.40	− 0.10	0.74
Knees (3 features), lateral left	Overall	31	n/a^a^	n/a^a^	n/a^a^
	Inflamed	18	n/a^a^	n/a^a^	n/a^a^
	Mixed	10	n/a^a^	n/a^a^	n/a^a^
	None	3	n/a^a^	n/a^a^	n/a^a^
Knees (3 features) lateral Right	Overall	30	0.29	− 0.08	0.59
	Inflamed	18	0.34	− 0.15	0.70
	Mixed	10	n/a^a^	n/a^a^	n/a^a^
	None	2	n/a^a^	n/a^a^	n/a^a^
Knees (3 features), medial left	Overall	31	0.30	− 0.06	0.59
	Inflamed	18	0.39	− 0.10	0.72
	Mixed	10	n/a^a^	n/a^a^	n/a^a^
	None	3	n/a^a^	n/a^a^	n/a^a^
Knees (3 features), medial right	Overall	30	0.36	0.01	0.64
	Inflamed	18	0.40	− 0.09	0.73
	Mixed	10	n/a^a^	n/a^a^	n/a^a^
	None	2	n/a^a^	n/a^a^	n/a^a^
Shoulder, ACJ left	Overall	52	0.56	0.34	0.73
	Inflamed	25	0.71	0.44	0.86
	Mixed	10	0.33	− 0.38	0.80
	None	17	0.45	− 0.04	0.77
Shoulder, ACJ right	Overall	53	0.61	0.41	0.76
	Inflamed	25	0.44	0.06	0.71
	Mixed	10	0.37	− 0.34	0.81
	None	18	0.69	0.32	0.87
Shoulder, GHJ left	Overall	39	0.26	− 0.06	0.53
	Inflamed	22	0.35	− 0.08	0.67
	Mixed	10	n/a^a^	n/a^a^	n/a^a^
	None	7	n/a^a^	n/a^a^	n/a^a^
Shoulder, GHJ right	Overall	39	0.24	− 0.08	0.52
	Inflamed	22	0.42	− 0.01	0.71
	Mixed	10	n/a^a^	n/a^a^	n/a^a^
	None	7	n/a^a^	n/a^a^	n/a^a^
Spine (3 features) cervical 1	Overall	51	0.27	− 0.01	0.51
	Inflamed	23	0.20	− 0.23	0.57
	Mixed	11	0.14	− 0.50	0.68
	None	17	0.42	− 0.07	0.75
Spine (3 features), cervical 2	Overall	51	0.38	0.11	0.59
	Inflamed	23	0.45	0.04	0.73
	Mixed	11	− 0.02	− 0.61	0.59
	None	17	0.55	0.10	0.82
Spine (3 features), lumbar 1	Overall	53	0.27	0.004	0.51
	Inflamed	25	0.30	− 0.11	0.62
	Mixed	11	0.31	− 0.36	0.77
	None	17	0.33	− 0.18	0.70
Spine (3 features), lumbar 2	Overall	54	0.42	0.17	0.62
	Inflamed	25	0.43	0.04	0.71
	Mixed	11	n/a^a^	n/a^a^	n/a^a^
	None	18	0.43	− 0.05	0.75
Spine (3 features), thoracic 1	Overall	52	0.23	− 0.05	0.47
	Inflamed	24	0.13	− 0.29	0.51
	Mixed	11	0.66	0.10	0.90
	None	17	− 0.05	− 0.52	0.44
Spine (3 features), thoracic 2	Overall	51	0.17	− 0.11	0.43
	Inflamed	24	0.24	− 0.18	0.58
	Mixed	11	0.15	− 0.50	0.69
	None	16	0.20	− 0.33	0.63

^a^No correlation analyses possible due to either low *n* in subgroup or no osteoarthritis observed

Concerning the reliability assessment, Table 3 gives an overview of inter- and intra-reader reliability for ordinal CT-based OA scoring based on w-kappa statistics and overall % agreement including 95% confidence intervals. Mean inter-reader reliability across all features and anatomies combined was 0.62 with overall percent agreement of 82.2%, and mean intra-reader reliability was 0.67 with overall percent agreement of 85.3%.

**Table 3:** Inter- and intra-reader reliability of semiquantitative osteoarthritis assessment on CT

Joint or structural feature^a^	Number of patients	Inter-reader reliability				Intra-reader reliability			
		W-kappa	**% **Agreement			W-kappa		% Agreement	
		Kappa	95% CI	Agreement	95% CI	Kappa	95% CI	Agreement	95% CI
Ankle	24	0.72	0.31, 1.00	91.7	0.79, 0.96	0.84	0.62, 1.00	95.8	0.88, 0.96
Elbows, JSW	14	1.00		100		1.00		100	
Elbows, osteophytes	14	1.00		100		1.00		100	
Hands, CMC D1	18	0.55	0.24, 0.86	77.8	0.50, 0.94	0.30	− 0.10, 0.71	72.2	0.50, 0.89
Hands, DIP D2–D5	68	0.60	0.39, 0.80	79.4	0.58, 0.94	0.83	0.69, 0.96	91.2	0.83, 0.98
Hands, IP D1	18	0.28	− 0.16, 0.72	61.1	0.33, 0.83	0.52	− 0.08, 1.00	83.3	0.67, 0.94
Hands, MCP D1	18	0.50	0.20, 0.80	66.7	0.44, 0.89	0.65	0.35, 0.94	77.8	0.61, 0.94
Hands, PIP D2–D5	70	0.39	0.15, 0.63	81.4	0.68, 0.93	0.72	0.53, 0.92	91.4	0.85, 0.97
Hip, cyst	30	0.63	0.18, 1.00	93.3	0.83, 0.97	0.63	0.18, 1.00	93.3	0.83, 0.97
Hip, JSW	30	0.51	0.07, 0.94	80.0	0.63, 0.93	0.60	0.28, 0.92	83.3	0.67, 0.97
Hip, osteophytes	30	0.88	0.63, 1.00	96.7	0.90, 0.97	0.48	0.00, 0.95	83.3	0.67, 0.97
Knee, cyst lateral	23	1.00		100	1.00, 1.00	1.00		100	
Knee, cyst medial	23	1.00		100	1.00, 1.00	1.00		100	
Knee, JSW lateral	23	1.00		100	1.00, 1.00	1.00		100	
Knee, JSW medial	23	0.66	0.38, 0.95	87.0	0.71, 0.96	0.60	0.21, 0.99	87.0	0.71, 0.96
Knee, osteophytes lateral	23	0.66	0.64, 0.68	95.7	0.83, 0.96	1.00	1.00, 1.00	100	
Knee, osteophytes medial	23	− 0.06	− 0.15, 0.02	87.0	0.65, 0.96	0.77	0.36, 1.00	95.7	0.86, 0.96
Shoulder, ACJ	30	0.56	0.30, 0.82	73.3	0.53, 0.90	0.58	0.34, 0.82	73.3	0.53, 0.90
Shoulder, GHJ	30	1.00	1.00, 1.00	100	1.00, 1.00	0.00	0.00, 0.00	96.7	0.87, 0.97
Spine, DSN cervical 1 and 2	38	0.55	0.34, 0.75	66.7	0.50, 0.83	0.73	0.55, 0.92	80.0	0.67, 0.93
Spine, DSN lumbar 1 and 2	30	0.54	0.20, 0.87	86.7	0.73, 0.97	0.68	0.39, 0.96	90.0	0.80, 0.97
Spine, DSN thoracic 1 and 2	30	0.24	− 0.08, 0.57	60.0	0.43, 0.77	0.40	0.06, 0.74	66.7	0.47, 0.83
Spine, OST cervical 1 and 2	30	0.70	0.52, 0.89	73.3	0.57, 0.87	0.72	0.54, 0.89	73.3	0.60, 0.85
Spine, OST lumbar 1 and 2	30	0.60	0.38, 0.82	63.3	0.47, 0.80	0.62	0.40, 0.84	66.7	0.48, 0.83
Spine, OST thoracic 1 and 2	30	0.75	0.57, 0.92	76.7	0.60, 0.90	0.68	0.48, 0.88	70.0	0.57, 0.83
Spine, SCIR cervical 1 and 2	30	0.61	0.39, 0.84	80.0	0.67, 0.93	0.50	0.25, 0.75	73.3	0.53, 0.90
Spine, SCIR lumbar 1 and 2	30	0.59	0.28, 0.91	83.3	0.70, 0.97	0.57	0.29, 0.85	80.0	0.67, 0.90
Spine, SCIR thoracic 1 and 2	30	− 0.02	− 0.31, 0.27	40.0	0.23, 0.57	0.29	0.01, 0.56	63.3	0.47, 0.80

Weighted kappa not calculable in locations with 100% observed agreement, and thus, cells are blank

*JSW* joint space width, *MCP* metacarpophalangeal joint, *OST* osteophytes

^a^Left and right combined for all peripheral joints

Regarding the quantitative SUV_max_ measurements, the large majority of joints and spine locations showed good or excellent reliability and high correlations for inter- as well as intra-reader assessment (Table 4) with maximum ICC values of up to 0.98 (hands PIP joints inter-reader or knee lateral intra-reader reliability). Low reliability was observed especially for the glenohumeral joint (inter-reader ICC − 0.04 and intra-reader ICC 0.11) and the IP D1 joint for inter-reader assessment (ICC 0.36).

**Table 4:** Inter- and intra-reader reliability of SUV_max_ determination per joint

Joint^a^ or anatomy	Number of patients	Inter-reader reliability		Intra-reader reliability	
		**ICC**	**Pearson correlation**		
Ankle	24	0.86	0.89	0.91	0.93
Elbows	14	0.97	0.98	0.98	0.98
Hands, CMC D1	18	0.66	0.69	0.50	0.52
Hands, DIP D2–D5	68	0.85	0.95	0.84	0.96
Hands, IP D1	18	0.36	0.35	0.85	0.87
Hands, MCP D1	18	0.53	0.51	0.95	0.96
Hands, PIP D2–D5	70	0.98	0.97	0.95	0.96
Hip	30	0.86	0.94	0.85	0.93
Knee, lateral	24	0.96	0.97	0.98	0.98
Knee, medial	24	0.97	0.97	0.92	0.93
Shoulder, ACJ	30	0.92	0.95	0.98	0.99
Shoulder, GHJ	30	− 0.04	0.19	0.11	0.24
Spine, cervical^b^	30	0.86	0.88	0.84	0.85
Spine, lumbar^b^	30	0.79	0.79	0.80	0.84
Spine, thoracic^b^	30	0.85	0.85	0.90	0.91

Reliability was assessed using the two-way mixed-effects, single-measures, absolute-agreement ICC (3,1)

^a^Left and right combined for peripheral joints

^b^Two regions of interest for each spine level

## Discussion

In this feasibility study using a convenience sample of patients who had undergone ^68^ Ga-FAPI PET-CT for a multitude of clinical reasons including assessment of inflammatory disease activity in a known rheumatologic inflammatory disorder or cancer staging, we found increased odds of high SUV_max_ FAPI uptake for both acromioclavicular joints, one cervical spine location, and both lumbar spine locations. In addition, we analyzed correlations of structural OA severity and FAPI uptake. Most correlations observed were small or medium with high correlations (> 0.5) seen for both acromioclavicular joints, left and right DIP, and right PIP joints for hands. Finally, we show that whole-body ordinal OA scoring from reformatted PET-CT images is feasible and can be performed with good reliability; quantitative SUV_max_ measurements can be obtained with a similarly high degree of reliability.

The literature on the potential role of FAPI PET and OA is sparse. A large single-center cohort study recently analyzed ^68^ Ga-FAPI PET scans in 268 patients who were scanned for oncologic reasons, correlating FAPI uptake in the acromioclavicular, glenohumeral, and hip joints with CT-based modified Kellgren–Lawrence OA grading [18]. The authors found that increased FAPI uptake in joints is common and can occur in both the presence and absence of radiographic OA. In that study, only in end-stage structural OA was FAPI uptake significantly and concordantly elevated. The study also demonstrated that FAPI uptake was heterogeneous in each Kellgren–Lawrence score group and that ranges overlapped, indicating a rather loose relationship between FAPI uptake and OA severity [18]. In comparison to that study, we included a more extensive analysis of large and small joints and also the spine, and at multiple levels. Furthermore, we used a more granular structural OA assessment beyond a simple composite (modified) Kellgren–Lawrence score and we performed extensive reliability exercises supporting the feasibility of whole-body ^68^ Ga-FAPI PET-CT assessment for detailed OA evaluation. In another study focusing on the shoulder joint, including 77 patients for diagnosis of solid tumors, Matsusaka and colleagues reported increased radiotracer accumulation in more than 43% of the subjects, in particular in the acromioclavicular joint [26]. While ^68^ Ga-FAPI-04 correlated with quantified ^18^F-FDG uptake, in vivo FAP expression was significantly increased relative to ^18^F-FDG.

Several studies have looked at ^68^ Ga-FAPI PET-CT in inflammatory disorders. Corte et al. conducted a prospective study looking at FAPI uptake and subsequent development of PsA and showed that patients with relevant synovial and entheseal ^68^ Ga-FAPI-04 uptake had a significantly higher risk of developing PsA independent of ultrasound findings [27]. These results suggest that the earliest signs in the joints and entheses of patients with psoriasis transitioning to PsA are hallmarked by the activation of tissue-resident mesenchymal cells and can be detected by ^68^ Ga-FAPI-04 PET-CT. In RA, FAPI PET-CT identifies more affected joints and demonstrates higher uptake than conventional ^18^F-FDG PET-CT, with uptake correlating strongly with clinical and laboratory markers of inflammation and radiographic progression [13]. A recent longitudinal study by Atzinger and co-workers further showed that synovial fibroblast activation occurs early in the disease process of RA and is associated with an increased risk of clinical RA onset [28]. In addition, FAPI PET data revealed a fundamental activation of fibroblasts in the joints of patients with different inflammatory disorders including RA, PsA, and axial spondylarthritis (axSpA), which was reversed by cytokine inhibition during resolution of disease [12]. For axSpA, Chen et al. showed that, compared to healthy volunteers, patients with axSpA exhibited significantly higher ^68^ Ga-FAPI-46 uptake in several joints, and especially in the spine [29]. ^68^ Ga-FAPI-46 PET-CT scores showed strong correlations with the Ankylosing Spondylitis Disease Activity Score, inflammatory indicators such as C-reactive protein, and inflammation visualized by MRI. Beyond RA and axSpA, FAPI PET-CT shows diagnostic value in other immune-mediated inflammatory diseases, including IgG4-related disease and Crohn’s disease, often outperforming FDG PET-CT in sensitivity and specificity for active inflammation and fibrotic burden [30, 31].

For ordinal assessment of OA, we used the OACT-Score as introduced by Gielis and colleagues, which provides detailed atlas-based guidance for semiquantitative scoring of large joints and spine including evaluation of osteophytes, sclerosis, joint space narrowing, or subchondral cyst formation [19]. For joints not described in the OACT atlas, we used a modified OARSI or Kellgren–Lawrence approach. Despite the primary reader being a junior researcher, we show that after extensive training and calibration, whole-body CT scoring is feasible with adequate reliability as shown by our inter- and intra-reader reliability data when compared to a very experienced musculoskeletal radiologist. We cannot rule out completely that the intra- and inter-reliability assessment may be different when comparing only highly specialized MSK radiologists with each other. Agreement is, however, influenced by many factors including but not limited to reader experience and training, frequency of features assessed, image quality, and other factors. In addition, ROI measurement for determining SUV_max_ could be performed with good reliability. We used SUV_max_ as an indicator of fibroblast activation and decided to not focus on SUV_mean_. SUV_max_ reflects the tracer uptake within the single hottest voxel in a given ROI, is not dependent on ROI size or shape, and thus reduces intra- and inter-observer variability [32]. A drawback of the ROI approach chosen, including the entire synovial joint or spine level, is based on the fact that adjacent extra-articular tissue pathology may interfere with determining the SUV_max_, such as periarticular tendon pathology in the form of tenosynovitis. We tried to include as little extra-articular pathology as possible by keeping ROIs optimized to the joint only, but cannot rule out some bias in this regard.

We used a modern PET-CT system and focused on FAPI-46 only while Spektor et al. used FAPI-46 and FAPI-74 in their study with no marked differences in the frequency and intensity of FAPI uptake [18]. Little previous work is available on types of FAPI tracer. Glatting et al. performed a structured comparison of FAPI-02, FAPI-46, and FAPI-74 uptake in malignant, inflammatory/reactive, and degenerative lesions [33]. Authors observed a higher uptake in inflammatory/reactive than in degenerative lesions for FAPI-46 and FAPI-74 with more pronounced differences for FAPI-46 than for FAPI-74 [18]. FAPI-02 showed similar SUVs in inflammatory/reactive and degenerative lesions, likely making FAPI-46 the tracer of choice for precise differentiation between inflammatory/reactive and degenerative findings such as OA [33].

Our study has several limitations. We used a convenience sample of patients who had undergone ^68^ Ga-FAPI PET for a multitude of reasons, which may limit the precision and generalizability of the findings. Given the exploratory nature of these analyses, we do not present *p* values. Confidence intervals were not adjusted for multiple testing and are presented primarily to convey the precision and uncertainty of the estimates. Cohen’s guidelines for interpreting correlation magnitude were originally developed for Pearson correlation coefficients; their application to Spearman correlations in this study, while common practice in the literature, has not been formally validated and should be interpreted with caution. We stratified our sample into three subgroups including one group without inflammatory disorder. Likely due to the relatively small sample size in each of the subgroups, we could not find fundamental differences between subgroups regarding associations or correlations of OA presence and severity and tracer uptake, although we did not analyze this systematically. Furthermore, we cannot definitely state that tracer uptake was due to OA or the underlying inflammatory disorder (for subgroups 1 and 2). The overall prevalence of OA and particularly severe OA in large joints was low, which might explain that associations and correlations between OA presence and severity and tracer uptake were modest and could be shown only for few of the joints and spine locations and, thus, makes interpretation regarding potential clinical implications challenging. In addition, mainly due to incomplete anatomical coverage, not all joints or spine locations could be assessed for all patients included. Furthermore, for reasons of feasibility, we did not include the facet joints, which are commonly affected by OA but also by inflammatory disease. In addition, we used only low-dose CT for attenuation correction. Furthermore, we are aware that there is a great difference in uptake time in the FAPI PET scans conducted in our cohort. However, previous work has shown that uptake time seems to have little effect on FAPI SUVs [34, 35].

Finally, the inherent limitations of CT and particularly its low soft tissue contrast do not allow for a direct comparison of FAPI tracer uptake with tissue-specific pathology such as synovitis, subchondral bone changes, or ligamentous or periligamentous pathology. These interactions will have to be explored in future work employing hybrid techniques such as PET-MRI that allow a more detailed anatomic assessment of soft tissue pathology. In addition, it needs mentioning that attenuation correction CT is not comparable to a diagnostic joint-focused CT regarding image quality, which may have also influenced the reliability results. Furthermore, the PET component was assessed by ROI analysis on a single reformatted slice. A spherical ROI or conforming ROI drawn to include all slices of a particular joint or anatomy—if technically feasible from an assessment standpoint—may be considered a superior reflection of SUV_max_ of radiotracer uptake and may improve intra- and inter-reader reliability assessment.

In summary, we show that presence of OA in the acromioclavicular joints was strongly associated with increased odds of SUV_max_ uptake. Increased odds were further seen for one location of the cervical spine and both locations of the lumbar spine. Moderate correlations were observed for OA severity and ^68^ Ga-FAPI PET uptake for most joints and spine. We further show that OA scoring and SUV_max_ determination from whole-body PET-CT can be performed with good reliability. Future longitudinal work will have to elucidate whether FAPI uptake actually precedes morphologic joint pathology and whether FAPI-positive and FAPI-negative (or only mildly positive) joints exhibit different trajectories of disease progression, which may have implications for future fibroblast-targeting therapeutic approaches.

## Supplementary Information

Below is the link to the electronic supplementary material.

ESM 1 (DOCX 72.8 KB)

## Data Availability

The data generated and analyzed during this study are available from the corresponding author upon reasonable request, subject to IRB approval and removal of personal health information.
