# Supplementary material for: Osteoarthritis and fibroblast activation: an imaging study assessing feasibility and reliability of whole-body 68 Ga-FAPI PET-CT for structural osteoarthritis evaluation
Source: Skeletal Radiol. 2026 Aug 31;55(12):3297–313. doi: 10.1007/s00256-026-05354-5 (PMC13669032; doi:10.1007/s00256-026-05354-5)
Supplement: Supplementary file 1 [file 256_2026_5354_MOESM1_ESM.docx]

**Table 1a.** Frequencies of structural osteoarthritis by joint and/or compartment and corresponding tracer uptake (SUV_max_): Large joints right

|  | | | | **All** | | | | | **Non-inflammation** | | | | | **Mixed** | | | | | **Inflammation** | | | | |
| --- | --- | --- | --- | --- | --- | --- | --- | --- | --- | --- | --- | --- | --- | --- | --- | --- | --- | --- | --- | --- | --- | --- | --- |
| **Side** |  | **Missing** | **Score** | **n** | **%** | **SUV Mean** | **SUV Min** | **SUV Max** | **n** | **%** | **SUV Mean** | **SUV Min** | **SUV Max** | **n** | **%** | **SUV Mean** | **SUV Min** | **SUV Max** | **n** | **%** | **SUV Mean** | **SUV Min** | **SUV Max** |
| Right | Shoulder – ACJ | 1 | 0 | 28 | 52.8% | 1.37 | 0.66 | 3.86 | 3 | 16.7% | 0.91 | 0.76 | 1.08 | 4 | 40.0% | 1.96 | 1.31 | 3.86 | 21 | 84.0% | 1.32 | 0.66 | 2.62 |
|  |  |  | 1 | 16 | 30.2% | 2.50 | 1.05 | 5.15 | 9 | 50.0% | 2.07 | 1.18 | 3.24 | 4 | 40.0% | 3.21 | 1.05 | 5.15 | 3 | 12.0% | 2.83 | 1.41 | 4.80 |
|  |  |  | 2 | 9 | 17.0% | 3.07 | 1.71 | 6.73 | 6 | 33.3% | 3.32 | 1.71 | 6.73 | 2 | 20.0% | 2.84 | 1.71 | 3.97 | 1 | 4.0% | 2.01 | 2.01 | 2.01 |
|  |  |  | 3 | 0 | 0.0% |  |  |  | 0 | 0.0% |  |  |  | 0 | 0.0% |  |  |  | 0 | 0.0% |  |  |  |
| Right | Shoulder – GHJ | 15 | 0 | 37 | 94.9% | 1.05 | 0.32 | 3.31 | 7 | 100% | 0.85 | 0.32 | 1.25 | 10 | 100% | 1.33 | 0.48 | 3.31 | 20 | 90.9% | 0.98 | 0.38 | 2.06 |
|  |  |  | 1 | 2 | 5.1% | 1.66 | 1.16 | 2.15 | 0 | 0.0% |  |  |  | 0 | 0.0% |  |  |  | 2 | 9.1% | 1.66 | 1.16 | 2.15 |
|  |  |  | 2 | 0 | 0.0% |  |  |  | 0 | 0.0% |  |  |  | 0 | 0.0% |  |  |  | 0 | 0.0% |  |  |  |
|  |  |  | 3 | 0 | 0.0% |  |  |  | 0 | 0.0% |  |  |  | 0 | 0.0% |  |  |  | 0 | 0.0% |  |  |  |
| Right | Elbow ^1^ | 36 | 0/1 no OA | 18 | 100% | 2.02 | 0.42 | 8.16 | 4 | 100% | 1.10 | 0.63 | 2.02 | 3 | 100% | 1.40 | 0.89 | 1.94 | 11 | 100% | 2.53 | 0.42 | 8.16 |
|  |  |  | 2/3 mild OA | 0 | 0.0% |  |  |  | 0 | 0.0% |  |  |  | 0 | 0.0% |  |  |  | 0 | 0.0% |  |  |  |
|  |  |  | 4-6 severe OA | 0 | 0.0% |  |  |  | 0 | 0.0% |  |  |  | 0 | 0.0% |  |  |  | 0 | 0.0% |  |  |  |
| Right | Hip ^2^ | 2 | Grade 0 (total score 0-1) | 48 | 92.3% | 1.31 | 0.07 | 4.40 | 16 | 94.1% | 1.51 | 0.59 | 4.40 | 10 | 90.9% | 1.08 | 0.07 | 2.61 | 22 | 91.7% | 1.27 | 0.27 | 4.05 |
|  |  |  | Grade 1 (total score 2-3) | 2 | 3.8% | 1.26 | 1.11 | 1.41 | 0 | 0.0% |  |  |  | 1 | 9.1% | 1.41 | 1.41 | 1.41 | 1 | 4.2% | 1.11 | 1.11 | 1.11 |
|  |  |  | Grade 2 (total score 4-5) | 2 | 3.8% | 8.19 | 5.15 | 11.23 | 1 | 5.9% | 5.15 | 5.15 | 5.15 | 0 | 0.0% |  |  |  | 1 | 4.2% | 11.23 | 11.23 | 11.23 |
|  |  |  | Grade 3 (total score 6-7) | 0 | 0.0% |  |  |  | 0 | 0.0% |  |  |  | 0 | 0.0% |  |  |  | 0 | 0.0% |  |  |  |
| Right | Knee lateral ^2^ | 24 | Grade 0 (total score 0-1) | 29 | 96.7% | 1.15 | 0.13 | 9.80 | 2 | 100% | 0.55 | 0.28 | 0.82 | 10 | 100% | 0.79 | 0.13 | 3.25 | 17 | 94.4% | 1.44 | 0.34 | 9.80 |
|  |  |  | Grade 1 (total score 2-3) | 1 | 3.3% | 6.83 | 6.83 | 6.83 | 0 | 0.0% |  |  |  | 0 | 0.0% |  |  |  | 1 | 5.6% | 6.83 | 6.83 | 6.83 |
|  |  |  | Grade 2 (total score 4-5) | 0 | 0.0% |  |  |  | 0 | 0.0% |  |  |  | 0 | 0.0% |  |  |  | 0 | 0.0% |  |  |  |
|  |  |  | Grade 3 (total score 6-7) | 0 | 0.0% |  |  |  | 0 | 0.0% |  |  |  | 0 | 0.0% |  |  |  | 0 | 0.0% |  |  |  |
| Right | Knee medial ^2^ | 24 | Grade 0 (total score 0-1) | 28 | 93.3% | 1.53 | 0.34 | 11.59 | 2 | 100% | 0.72 | 0.46 | 0.98 | 10 | 100% | 1.33 | 0.37 | 3.19 | 16 | 88.9% | 1.76 | 0.34 | 11.59 |
|  |  |  | Grade 1 (total score 2-3) | 1 | 3.3% | 6.78 | 6.78 | 6.78 | 0 | 0.0% |  |  |  | 0 | 0.0% |  |  |  | 1 | 5.6% | 6.78 | 6.78 | 6.78 |
|  |  |  | Grade 2 (total score 4-5) | 1 | 3.3% | 5.22 | 5.22 | 5.22 | 0 | 0.0% |  |  |  | 0 | 0.0% |  |  |  | 1 | 5.6% | 5.22 | 5.22 | 5.22 |
|  |  |  | Grade 3 (total score 6-7) | 0 | 0.0% |  |  |  | 0 | 0.0% |  |  |  | 0 | 0.0% |  |  |  | 0 | 0.0% |  |  |  |
| Right | Ankle | 26 | Grade 0 | 28 | 96.6% | 1.04 | 0.23 | 9.81 | 3 | 100% | 0.50 | 0.34 | 0.63 | 9 | 90.0% | 0.80 | 0.30 | 2.58 | 16 | 100% | 1.28 | 0.23 | 9.81 |
|  |  |  | Grade 1 | 0 | 0.0% |  |  |  | 0 | 0.0% |  |  |  | 0 | 0.0% |  |  |  | 0 | 0.0% |  |  |  |
|  |  |  | Grade 2 | 1 | 3.4% | 2.23 | 2.23 | 2.23 | 0 | 0.0% |  |  |  | 1 | 10.0% | 2.23 | 2.23 | 2.23 | 0 | 0.0% |  |  |  |
|  |  |  | Grade 3 | 0 | 0.0% |  |  |  | 0 | 0.0% |  |  |  | 0 | 0.0% |  |  |  | 0 | 0.0% |  |  |  |

ACJ – acromioclavicular joint; GHJ – glenohumeral joint; SUV – standardized uptake value (SUV in all supplementary Tables 1-3 means SUV_max_)

^1^ Two features scored: osteophytes and joint space width; ^2^ Three features scored: osteophytes, joint space width, subchondral cysts

**Table 1b.** Frequencies of structural osteoarthritis by joint and/or compartment and corresponding tracer uptake (SUV_max_): Large joints left

|  | | | | **All** | | | | | **Non-inflammation** | | | | | **Mixed** | | | | | **Inflammation** | | | | |
| --- | --- | --- | --- | --- | --- | --- | --- | --- | --- | --- | --- | --- | --- | --- | --- | --- | --- | --- | --- | --- | --- | --- | --- |
| **side** |  | **missing** | **Score** | **n** | **%** | **SVU Mean** | **SVU Min** | **SVU Max** | **n** | **%** | **SVU Mean** | **SVU Min** | **SVU Max** | **n** | **%** | **SVU Mean** | **SVU Min** | **SVU Max** | **n** | **%** | **SVU Mean** | **SVU Min** | **SVU Max** |
| Left | Shoulder – ACJ | 2 | 0 | 21 | 40.4% | 1.26 | 0.77 | 3.22 | 3 | 17.6% | 0.97 | 0.91 | 1.03 | 5 | 50.0% | 2.10 | 1.05 | 3.22 | 13 | 52.0% | 1.01 | 0.77 | 1.18 |
|  |  |  | 1 | 24 | 46.2% | 2.42 | 0.73 | 5.82 | 9 | 52.9% | 2.21 | 1.20 | 3.30 | 5 | 50.0% | 3.62 | 0.73 | 5.82 | 10 | 40.0% | 2.01 | 0.87 | 3.59 |
|  |  |  | 2 | 7 | 13.5% | 2.62 | 1.21 | 4.67 | 5 | 29.4% | 2.43 | 1.21 | 4.67 | 0 | 0.0% |  |  |  | 2 | 8.0% | 3.11 | 1.83 | 4.38 |
|  |  |  | 3 | 0 | 0.0% |  |  |  | 0 | 0.0% |  |  |  | 0 | 0.0% |  |  |  | 0 | 0.0% |  |  |  |
| Left | Shoulder – GHJ | 15 | 0 | 38 | 97.4% | 1.10 | 0.36 | 2.99 | 7 | 100% | 0.95 | 0.65 | 1.31 | 10 | 100% | 1.42 | 0.61 | 2.99 | 21 | 95.5% | 0.99 | 0.36 | 1.40 |
|  |  |  | 1 | 1 | 2.6% | 2.42 | 2.42 | 2.42 | 0 | 0.0% |  |  |  | 0 | 0.0% |  |  |  | 1 | 4.5% | 2.42 | 2.42 | 2.42 |
|  |  |  | 2 | 0 | 0.0% |  |  |  | 0 | 0.0% |  |  |  | 0 | 0.0% |  |  |  | 0 | 0.0% |  |  |  |
|  |  |  | 3 | 0 | 0.0% |  |  |  | 0 | 0.0% |  |  |  | 0 | 0.0% |  |  |  | 0 | 0.0% |  |  |  |
| Left | Elbow ^1^ | 35 | 0/1 no OA | 19 | 100% | 1.17 | 0.49 | 2.22 | 5 | 100% | 1.52 | 0.87 | 2.22 | 2 | 100% | 0.99 | 0.70 | 1.27 | 12 | 100% | 1.05 | 0.49 | 1.92 |
|  |  |  | 2/3 mild OA | 0 | 0.0% |  |  |  | 0 | 0.0% |  |  |  | 0 | 0.0% |  |  |  | 0 | 0.0% |  |  |  |
|  |  |  | 4-6 severe OA | 0 | 0.0% |  |  |  | 0 | 0.0% |  |  |  | 0 | 0.0% |  |  |  | 0 | 0.0% |  |  |  |
| Left | Hips ^2^ | 2 | Grade 0 (total score 0-1) | 50 | 96.2% | 1.19 | 0.06 | 2.94 | 16 | 94.1% | 1.22 | 0.54 | 2.13 | 11 | 100% | 1.15 | 0.06 | 2.94 | 23 | 95.8% | 1.20 | 0.31 | 2.79 |
|  |  |  | Grade 1 (total score 2-3) | 1 | 1.9% | 4.99 | 4.99 | 4.99 | 1 | 5.9% | 4.99 | 4.99 | 4.99 | 0 | 0.0% |  |  |  | 0 | 0.0% |  |  |  |
|  |  |  | Grade 2 (total score 4-5) | 1 | 1.9% | 6.03 | 6.03 | 6.03 | 0 | 0.0% |  |  |  | 0 | 0.0% |  |  |  | 1 | 4.2% | 6.03 | 6.03 | 6.03 |
|  |  |  | Grade 3 (total score 6-7) | 0 | 0.0% |  |  |  | 0 | 0.0% |  |  |  | 0 | 0.0% |  |  |  | 0 | 0.0% |  |  |  |
| Left | Knee lateral ^2^ | 23 | Grade 0 (total score 0-1) | 31 | 100% | 1.33 | 0.28 | 13.01 | 3 | 100% | 0.59 | 0.35 | 0.96 | 10 | 100% | 1.08 | 0.36 | 4.79 | 18 | 100% | 1.59 | 0.28 | 13.01 |
|  |  |  | Grade 1 (total score 2-3) | 0 | 0.0% |  |  |  | 0 | 0.0% |  |  |  | 0 | 0.0% |  |  |  | 0 | 0.0% |  |  |  |
|  |  |  | Grade 2 (total score 4-5) | 0 | 0.0% |  |  |  | 0 | 0.0% |  |  |  | 0 | 0.0% |  |  |  | 0 | 0.0% |  |  |  |
|  |  |  | Grade 3 (total score 6-7) | 0 | 0.0% |  |  |  | 0 | 0.0% |  |  |  | 0 | 0.0% |  |  |  | 0 | 0.0% |  |  |  |
| Left | Knee medial ^2^ | 23 | Grade 0 (total score 0-1) | 30 | 96.8% | 0.93 | 0.30 | 6.47 | 3 | 100% | 0.66 | 0.39 | 0.98 | 10 | 100% | 1.18 | 0.43 | 6.47 | 17 | 94.4% | 0.82 | 0.30 | 2.63 |
|  |  |  | Grade 1 (total score 2-3) | 1 | 3.2% | 15.85 | 15.85 | 15.85 | 0 | 0.0% |  |  |  | 0 | 0.0% |  |  |  | 1 | 5.6% | 15.85 | 15.85 | 15.85 |
|  |  |  | Grade 2 (total score 4-5) | 0 | 0.0% |  |  |  | 0 | 0.0% |  |  |  | 0 | 0.0% |  |  |  | 0 | 0.0% |  |  |  |
|  |  |  | Grade 3 (total score 6-7) | 0 | 0.0% |  |  |  | 0 | 0.0% |  |  |  | 0 | 0.0% |  |  |  | 0 | 0.0% |  |  |  |
| Left | Ankle | 25 | Grade 0 | 27 | 93.1% | 0.98 | 0.23 | 12.84 | 2 | 66.7% | 0.37 | 0.33 | 0.40 | 10 | 100% | 0.48 | 0.25 | 1.06 | 15 | 93.8% | 1.40 | 0.23 | 12.84 |
|  |  |  | Grade 1 | 2 | 6.9% | 1.77 | 0.45 | 3.08 | 1 | 33.3% | 0.45 | 0.45 | 0.45 | 0 | 0.0% |  |  |  | 1 | 6.3% | 3.08 | 3.08 | 3.08 |
|  |  |  | Grade 2 | 0 | 0.0% |  |  |  | 0 | 0.0% |  |  |  | 0 | 0.0% |  |  |  | 0 | 0.0% |  |  |  |
|  |  |  | Grade 3 | 0 | 0.0% |  |  |  | 0 | 0.0% |  |  |  | 0 | 0.0% |  |  |  | 0 | 0.0% |  |  |  |

ACJ – acromioclavicular joint; GHJ – glenohumeral joint; SUV – standardized uptake value

^1^ Two features scored: osteophytes and joint space width; ^2^ Three features scored: osteophytes joint space width, subchondral cysts

**Table 2a.** Frequencies of structural osteoarthritis by joint and/or compartment and corresponding tracer uptake (SUV_max_): Hands right

|  | | | | **All** | | | | | **Non-inflammation** | | | | | **Mixed** | | | | | | **Inflammation** | | | | |
| --- | --- | --- | --- | --- | --- | --- | --- | --- | --- | --- | --- | --- | --- | --- | --- | --- | --- | --- | --- | --- | --- | --- | --- | --- |
| **side** |  | **Missing** | **Score** | **n** | **%** | **SUV Mean** | **SUV Min** | **SUV Max** | **n** | **%** | **SUV Mean** | **SUV Min** | **SUV Max** | **n** | | **%** | **SUV Mean** | **SUV Min** | **SUV Max** | **n** | **%** | **SUV Mean** | **SUV Min** | **SUV Max** |
| Right | Hands – CMC D1 | 32 | 0 | 19 | 86.4% | 1.42 | 0.06 | 2.96 | 0 | 0.0% |  |  |  | 8 | 88.9% | | 1.40 | 0.06 | 2.16 | 11 | 91.7% | 1.42 | 0.90 | 2.96 |
|  |  |  | 1 | 3 | 13.6% | 1.13 | 0.73 | 1.94 | 1 | 100% | 1.94 | 1.94 | 1.94 | 1 | 11.1% | | 0.73 | 0.73 | 0.73 | 1 | 8.3% | 0.73 | 0.73 | 0.73 |
|  |  |  | 2 | 0 | 0.0% |  |  |  | 0 | 0.0% |  |  |  | 0 | 0.0% | |  |  |  | 0 | 0.0% |  |  |  |
|  |  |  | 3 | 0 | 0.0% |  |  |  | 0 | 0.0% |  |  |  | 0 | 0.0% | |  |  |  | 0 | 0.0% |  |  |  |
| Right | Hands – IP joint D1 | 33 | 0 | 18 | 85.7% | 0.98 | 0.37 | 2.49 | 1 | 100% | 2.49 | 2.49 | 2.49 | 5 | 62.5% | | 1.19 | 0.49 | 2.07 | 12 | 100% | 0.76 | 0.37 | 1.40 |
|  |  |  | 1 | 3 | 14.3% | 1.12 | 0.87 | 1.29 | 0 | 0.0% |  |  |  | 3 | 37.5% | | 1.12 | 0.87 | 1.29 | 0 | 0.0% |  |  |  |
|  |  |  | 2 | 0 | 0.0% |  |  |  | 0 | 0.0% |  |  |  | 0 | 0.0% | |  |  |  | 0 | 0.0% |  |  |  |
|  |  |  | 3 | 0 | 0.0% |  |  |  | 0 | 0.0% |  |  |  | 0 | 0.0% | |  |  |  | 0 | 0.0% |  |  |  |
| Right | Hands – DIP joints ^1^ | 41 | 0 | 5 | 38.5% | 1.37 | 0.68 | 1.98 | 0 | 0.0% |  |  |  | 3 | 42.9% | | 1.78 | 1.58 | 1.98 | 2 | 40.0% | 0.77 | 0.68 | 0.85 |
|  |  |  | 1 | 3 | 23.1% | 2.18 | 2.13 | 2.25 | 0 | 0.0% |  |  |  | 1 | 14.3% | | 2.25 | 2.25 | 2.25 | 2 | 40.0% | 2.15 | 2.13 | 2.17 |
|  |  |  | 2 | 2 | 15.4% | 2.07 | 2.00 | 2.13 | 1 | 100% | 2.00 | 2.00 | 2.00 | 0 | 0.0% | |  |  |  | 1 | 20.0% | 2.13 | 2.13 | 2.13 |
|  |  |  | 3+ | 3 | 23.1% | 3.08 | 1.81 | 5.43 | 0 | 0.0% |  |  |  | 3 | 42.9% | | 3.08 | 1.81 | 5.43 | 0 | 0.0% |  |  |  |
| Right | Hands – PIP joints ^1^ | 37 | 0 | 11 | 64.7% | 1.43 | 0.47 | 3.79 | 0 | 0.0% |  |  |  | 6 | 75.0% | | 1.44 | 1.06 | 1.97 | 5 | 62.5% | 1.41 | 0.47 | 3.79 |
|  |  |  | 1 | 2 | 11.8% | 1.31 | 0.91 | 1.71 | 0 | 0.0% |  |  |  | 0 | 0.0% | |  |  |  | 2 | 25.0% | 1.31 | 0.91 | 1.71 |
|  |  |  | 2 | 3 | 17.6% | 2.88 | 1.98 | 3.51 | 1 | 100% | 1.98 | 1.98 | 1.98 | 2 | 25.0% | | 3.34 | 3.16 | 3.51 | 0 | 0.0% |  |  |  |
|  |  |  | 3+ | 1 | 5.9% | 3.57 | 3.57 | 3.57 | 0 | 0.0% |  |  |  | 0 | 0.0% | |  |  |  | 1 | 12.5% | 3.57 | 3.57 | 3.57 |

CMC -carpometacarpal joint; IP -interpahalangeal; DIP – distal interphalangeal; PIP – proximal interphalangeal

^1^ two features scored: osteophytes joint space width (summed range 0-6)

**Table 2b.** Frequencies of structural osteoarthritis by joint and/or compartment and corresponding tracer uptake (SUV_max_): Hands left

|  | | | | **All** | | | | | **Non-inflammation** | | | | | **Mixed** | | | | | **Inflammation** | | | | |
| --- | --- | --- | --- | --- | --- | --- | --- | --- | --- | --- | --- | --- | --- | --- | --- | --- | --- | --- | --- | --- | --- | --- | --- |
| **side** |  | **MIssing** | **Score** | **n** | **%** | **SUV Mean** | **SUV Min** | **SUV Max** | **n** | **%** | **SUV Mean** | **SUV Min** | **SUV Max** | **n** | **%** | **SUV Mean** | **SUV Min** | **SUV Max** | **n** | **%** | **SUV Mean** | **SUV Min** | **SUV Max** |
| Left | Hands – CMC D1 | 32 | 0 | 18 | 81.8% | 1.61 | 0.70 | 4.19 | 1 | 100% | 1.64 | 1.64 | 1.64 | 7 | 77.8% | 1.74 | 1.11 | 4.19 | 10 | 83.3% | 1.52 | 0.70 | 3.25 |
|  |  |  | 1 | 3 | 13.6% | 1.53 | 1.27 | 1.74 | 0 | 0.0% |  |  |  | 1 | 11.1% | 1.27 | 1.27 | 1.27 | 2 | 16.7% | 1.66 | 1.58 | 1.74 |
|  |  |  | 2 | 1 | 4.5% | 2.49 | 2.49 | 2.49 | 0 | 0.0% |  |  |  | 1 | 11.1% | 2.49 | 2.49 | 2.49 | 0 | 0.0% |  |  |  |
|  |  |  | 3 | 0 | 0.0% |  |  |  | 0 | 0.0% |  |  |  | 0 | 0.0% |  |  |  | 0 | 0.0% |  |  |  |
| Left | Hands – IP joint D1 | 33 | 0 | 19 | 90.5% | 1.16 | 0.26 | 1.68 | 1 | 100% | 1.33 | 1.33 | 1.33 | 7 | 87.5% | 1.31 | 0.74 | 1.62 | 11 | 91.7% | 1.03 | 0.26 | 1.68 |
|  |  |  | 1 | 1 | 4.8% | 0.99 | 0.99 | 0.99 | 0 | 0.0% |  |  |  | 1 | 12.5% | 0.99 | 0.99 | 0.99 | 0 | 0.0% |  |  |  |
|  |  |  | 2 | 1 | 4.8% | 2.89 | 2.89 | 2.89 | 0 | 0.0% |  |  |  | 0 | 0.0% |  |  |  | 1 | 8.3% | 2.89 | 2.89 | 2.89 |
|  |  |  | 3 | 0 | 0.0% |  |  |  | 0 | 0.0% |  |  |  | 0 | 0.0% |  |  |  | 0 | 0.0% |  |  |  |
| Left | Hands – DIP joints | 38 | 0 | 7 | 43.8% | 1.28 | 0.59 | 2.11 | 0 | 0.0% |  |  |  | 4 | 50.0% | 1.64 | 1.02 | 2.11 | 3 | 42.9% | 0.79 | 0.59 | 0.94 |
|  |  |  | 1 | 4 | 25.0% | 2.58 | 1.20 | 4.78 | 0 | 0.0% |  |  |  | 1 | 12.5% | 4.78 | 4.78 | 4.78 | 3 | 42.9% | 1.84 | 1.20 | 2.35 |
|  |  |  | 2 | 4 | 25.0% | 2.56 | 2.22 | 3.28 | 1 | 100% | 2.26 | 2.26 | 2.26 | 2 | 25.0% | 2.89 | 2.49 | 3.28 | 1 | 14.3% | 2.22 | 2.22 | 2.22 |
|  |  |  | 3+ | 1 | 6.3% | 4.69 | 4.69 | 4.69 | 0 | 0.0% |  |  |  | 1 | 12.5% | 4.69 | 4.69 | 4.69 | 0 | 0.0% |  |  |  |
| Left | Hands – PIP joints | 37 | 0 | 9 | 52.9% | 1.68 | 0.46 | 5.84 | 0 | 0.0% |  |  |  | 4 | 50.0% | 1.39 | 0.83 | 1.85 | 5 | 62.5% | 1.91 | 0.46 | 5.84 |
|  |  |  | 1 | 6 | 35.3% | 1.70 | 0.97 | 2.36 | 1 | 100% | 2.27 | 2.27 | 2.27 | 3 | 37.5% | 1.77 | 0.98 | 2.36 | 2 | 25.0% | 1.31 | 0.97 | 1.64 |
|  |  |  | 2 | 1 | 5.9% | 1.02 | 1.02 | 1.02 | 0 | 0.0% |  |  |  | 0 | 0.0% |  |  |  | 1 | 12.5% | 1.02 | 1.02 | 1.02 |
|  |  |  | 3+ | 1 | 5.9% | 5.63 | 5.63 | 5.63 | 0 | 0.0% |  |  |  | 1 | 12.5% | 5.63 | 5.63 | 5.63 | 0 | 0.0% |  |  |  |

CMC -carpometacarpal joint; IP -interpahalangeal; DIP – distal interphalangeal; PIP – proximal interphalangeal

^1^ two features scored: osteophytes joint space width (summed range 0-6)

**Table 3.** Frequencies of structural osteoarthritis and corresponding tracer uptake (SUV_max_): Spine

|  | | | **All** | | | | | **Non-inflammation** | | | | | **Mixed** | | | | | **Inflammation** | | | | |
| --- | --- | --- | --- | --- | --- | --- | --- | --- | --- | --- | --- | --- | --- | --- | --- | --- | --- | --- | --- | --- | --- | --- |
| **Anatomy/Level** | **Missing** | **Score** | **n** | **%** | **SUV Mean** | **SUV Min** | **SUV Max** | **n** | **%** | **SUV Mean** | **SUV Min** | **SUV Max** | **n** | **%** | **SUV Mean** | **SUV Min** | **SUV Max** | **n** | **%** | **SUV Mean** | **SUV Min** | **SUV Max** |
| Spine – cervical 1 ^1^ | 3 | Grade 0 (total score 0-2) | 35 | 68.6% | 1.37 | 0.61 | 3.04 | 8 | 47.1% | 1.12 | 0.71 | 1.71 | 8 | 72.7% | 1.48 | 0.61 | 3.04 | 19 | 82.6% | 1.44 | 0.64 | 2.71 |
|  |  | Grade 1 (total score 3-5) | 10 | 19.6% | 2.27 | 0.73 | 4.64 | 6 | 35.3% | 2.53 | 0.76 | 4.64 | 2 | 18.2% | 1.57 | 0.73 | 2.41 | 2 | 8.7% | 2.18 | 1.11 | 3.24 |
|  |  | Grade 2 (total score 6-8) | 6 | 11.8% | 1.90 | 0.86 | 2.70 | 3 | 17.6% | 1.93 | 0.86 | 2.52 | 1 | 9.1% | 1.74 | 1.74 | 1.74 | 2 | 8.7% | 1.93 | 1.15 | 2.70 |
|  |  | Grade 3 (total score 9-10 | 0 | 0.0% |  |  |  | 0 | 0.0% |  |  |  | 0 | 0.0% |  |  |  | 0 | 0.0% |  |  |  |
| Spine – cervical 2 | 3 | Grade 0 (total score 0-2) | 24 | 47.1% | 1.32 | 0.37 | 2.86 | 5 | 29.4% | 1.31 | 1.03 | 1.98 | 4 | 36.4% | 1.78 | 1.14 | 2.86 | 15 | 65.2% | 1.20 | 0.37 | 2.08 |
|  |  | Grade 1 (total score 3-5) | 19 | 37.3% | 1.54 | 0.43 | 3.44 | 7 | 41.2% | 1.41 | 1.24 | 1.64 | 6 | 54.5% | 1.73 | 0.43 | 3.44 | 6 | 26.1% | 1.50 | 1.00 | 2.34 |
|  |  | Grade 2 (total score 6-8) | 8 | 15.7% | 2.14 | 1.12 | 3.69 | 5 | 29.4% | 1.99 | 1.12 | 2.81 | 1 | 9.1% | 1.69 | 1.69 | 1.69 | 2 | 8.7% | 2.71 | 1.73 | 3.69 |
|  |  | Grade 3 (total score 9-10 | 0 | 0.0% |  |  |  | 0 | 0.0% |  |  |  | 0 | 0.0% |  |  |  | 0 | 0.0% |  |  |  |
| Spine – thoracic 1 | 2 | Grade 0 (total score 0-2) | 20 | 38.5% | 1.30 | 0.41 | 3.06 | 6 | 35.3% | 1.43 | 0.41 | 3.06 | 4 | 36.4% | 0.97 | 0.44 | 1.51 | 10 | 41.7% | 1.36 | 0.78 | 2.06 |
|  |  | Grade 1 (total score 3-5) | 27 | 51.9% | 1.55 | 0.67 | 3.59 | 10 | 58.8% | 1.44 | 0.67 | 2.18 | 5 | 45.5% | 1.85 | 1.02 | 3.59 | 12 | 50.0% | 1.50 | 0.89 | 2.42 |
|  |  | Grade 2 (total score 6-8) | 5 | 9.6% | 1.71 | 0.64 | 2.83 | 1 | 5.9% | 0.64 | 0.64 | 0.64 | 2 | 18.2% | 2.49 | 2.15 | 2.83 | 2 | 8.3% | 1.47 | 0.99 | 1.95 |
|  |  | Grade 3 (total score 9-10 | 0 | 0.0% |  |  |  | 0 | 0.0% |  |  |  | 0 | 0.0% |  |  |  | 0 | 0.0% |  |  |  |
| Spine – thoracic 2 | 3 | Grade 0 (total score 0-2) | 22 | 43.1% | 1.35 | 0.80 | 3.23 | 7 | 43.8% | 1.30 | 1.00 | 1.66 | 6 | 54.5% | 1.62 | 0.93 | 3.23 | 9 | 37.5% | 1.21 | 0.80 | 1.94 |
|  |  | Grade 1 (total score 3-5) | 26 | 51.0% | 1.60 | 0.48 | 3.56 | 8 | 50.0% | 1.60 | 0.48 | 3.56 | 4 | 36.4% | 1.90 | 0.60 | 3.01 | 14 | 58.3% | 1.52 | 0.75 | 2.88 |
|  |  | Grade 2 (total score 6-8) | 3 | 5.9% | 1.96 | 1.37 | 2.86 | 1 | 6.3% | 2.86 | 2.86 | 2.86 | 1 | 9.1% | 1.37 | 1.37 | 1.37 | 1 | 4.2% | 1.65 | 1.65 | 1.65 |
|  |  | Grade 3 (total score 9-10 | 0 | 0.0% |  |  |  | 0 | 0.0% |  |  |  | 0 | 0.0% |  |  |  | 0 | 0.0% |  |  |  |
| Spine – lumbar 1 | 1 | Grade 0 (total score 0-2) | 45 | 84.9% | 1.37 | 0.41 | 3.50 | 14 | 82.4% | 1.33 | 0.41 | 2.89 | 8 | 72.7% | 1.16 | 0.68 | 1.60 | 23 | 92.0% | 1.47 | 0.89 | 3.50 |
|  |  | Grade 1 (total score 3-5) | 6 | 11.3% | 1.43 | 0.89 | 1.75 | 2 | 11.8% | 1.36 | 1.18 | 1.54 | 3 | 27.3% | 1.38 | 0.89 | 1.75 | 1 | 4.0% | 1.73 | 1.73 | 1.73 |
|  |  | Grade 2 (total score 6-8) | 2 | 3.8% | 2.56 | 1.56 | 3.56 | 1 | 5.9% | 3.56 | 3.56 | 3.56 | 0 | 0.0% |  |  |  | 1 | 4.0% | 1.56 | 1.56 | 1.56 |
|  |  | Grade 3 (total score 9-10 | 0 | 0.0% |  |  |  | 0 | 0.0% |  |  |  | 0 | 0.0% |  |  |  | 0 | 0.0% |  |  |  |
| Spine – lumbar 2 | 0 | Grade 0 (total score 0-2) | 41 | 75.9% | 1.26 | 0.31 | 2.56 | 10 | 55.6% | 1.26 | 1.00 | 2.24 | 11 | 100% | 1.15 | 0.31 | 1.92 | 20 | 80.0% | 1.31 | 0.67 | 2.56 |
|  |  | Grade 1 (total score 3-5) | 10 | 18.5% | 1.97 | 0.95 | 4.41 | 6 | 33.3% | 1.86 | 0.95 | 4.41 | 0 | 0.0% |  |  |  | 4 | 16.0% | 2.12 | 1.66 | 2.44 |
|  |  | Grade 2 (total score 6-8) | 3 | 5.6% | 1.90 | 1.25 | 2.63 | 2 | 11.1% | 2.23 | 1.83 | 2.63 | 0 | 0.0% |  |  |  | 1 | 4.0% | 1.25 | 1.25 | 1.25 |
|  |  | Grade 3 (total score 9-10 | 0 | 0.0% |  |  |  | 0 | 0.0% |  |  |  | 0 | 0.0% |  |  |  | 0 | 0.0% |  |  |  |

^1^ Three features scored: disc space narrowing, osteophytes, sclerosis and irregularity
